# Safety of intravitreal ziv-aflibercept in choroido-retinal vascular diseases: A randomised double-blind intervention study

**DOI:** 10.1371/journal.pone.0223944

**Published:** 2019-10-24

**Authors:** Imoro Zeba Braimah, Ernest Kenu, Kwesi N. Amissah-Arthur, Stephen Akafo, Kwaku Oppong Kwarteng, Winfried M. Amoaku

**Affiliations:** 1 Department of Surgery (Eye), School of Medicine and Dentistry, College of Health Sciences, University of Ghana, Accra, Ghana; 2 Eye Centre, Korle- Bu Teaching Hospital, Korle- Bu, Accra, Ghana; 3 Department of Epidemiology, School of Public Health, University of Ghana, Accra, Ghana; 4 Academic Ophthalmology, DCN, Faculty of Medicine and Health Sciences, University of Nottingham, Nottingham, England, United Kingdom; Universita degli Studi di Firenze, ITALY

## Abstract

**Aim:**

To evaluate the safety of 1.25mg and 2mg intravitreal ziv-aflibercept (IVZ) in Ghanaian eyes with choroido-retinal vascular diseases.

**Design:**

Prospective, randomised, double blind, interventional study.

**Methods:**

Twenty patients with centre involving macular oedema in diabetic retinopathy, retinal vein occlusion, and neovascular age-related macular degeneration were assigned to 2 groups receiving 3 doses of 1.25mg/0.05ml (group 1) and 2mg/0.08ml IVZ (Group 2) at 4 weekly intervals. Safety data was collected after 30 minutes, 1 and 7 days, and 4, 8 and 12 weeks after injection. Changes in continuous variables were compared using paired t-test and categorical variables were compared using chi-square test of proportions. Repeated-Measures ANOVA with nesting test was used to compare variations in continuous variables by IVZ dose over time. Primary outcome measures were ocular and systemic adverse events at 4 weeks.

**Results:**

Eleven females and nine males, with mean age of 63.2± 7.3 years were included. Ocular adverse events included subconjunctival haemorrhage in 1 eye, intraocular pressure (IOP) >21mmHg at 30 minutes in 6 eyes and mild pain in 3 eyes at 1-day. There was no significant difference in IOP rise between the 2 groups at 30 minutes (p = 0.21). No other ocular or systemic adverse events were observed. There was significant improvement in the best corrected visual acuity (LogMAR) from 0.95±0.6 to 0.6±0.4 (p<0.01) and 0.47±0.3 (p<0.01), reduction in central subfield foveal thickness from 405.9±140 um at baseline to 255.6±75 um (p<0.01) and 238±88 um (p<0.01) at 4 and 12 weeks respectively, although no difference was observed between the 2 groups (p = 0.34).

**Conclusion:**

IVZ at 1.25mg and 2mg had similar safety profiles, and did not have any major unexpected adverse events. Further studies with larger cohorts are required to confirm efficacy.

## Introduction

Retinal and choroidal vascular diseases such as diabetic macular oedema (DMO), neovascular age-related macular (nvAMD), and macular oedema (MO) following retinal vein occlusions (RVO) are a significant cause of visual impairment in developed countries. They are increasingly becoming important causes of blindness in developing and low-middle income countries including Ghana [1–3]. Vascular endothelial growth factor, which promotes angiogenesis and increases vascular permeability, has been found to play an important role in the pathogenesis of these diseases [4–6]. Ranibizumab (Lucentis; Genentech, San Francisco, California, USA/Novartis, Basel, Switzerland) and aflibercept (Eylea; Regeneron, Tarrytown, New York, USA) have been approved by the USA Food and Drugs Agency (FDA) and European Medicines Agency (EMA) for the treatment of DMO, nvAMD and MO following RVO. Bevacizumab (Avastin, Genentech Inc. USA /Roche, Basel, Switzerland) and ziv-aflibercept (Zaltrap, Sanofi-Aventis US, LLC, Bridgewater, New Jersey, USA and Regeneron Pharmaceuticals, Inc., Tarrytown, New York, USA) have been approved by the US FDA and EMA for the treatment of colorectal cancers [7]. Unfortunately, ranibizumab and aflibercept are expensive (US $1950 and $1850 per dose respectively) and many needy patients in developing countries including Ghana lack the funds to pay for these treatments which are self-funded. The unavailability of rebranded equivalents for poorer countries has compounded the problems in sub-Saharan Africa.

Bevacizumab (off-label use) is the most commonly used anti-VEGF worldwide. This is particularly so in the developing world, where its cost-effectiveness when compounded is of economic benefit in the treatment of retinal vascular diseases [8]. Due to individual variability in their response to a particular anti-VEGF the availability of alternative agents that are of similar cost to bevacizumab will be useful particularly in patients who are recalcitrant or ‘poor responders’ or ‘non-responders’ to bevacizumab [9]. Short-term and a few long-term reports have shown that the 1.25 mg dose of ziv-aflibercept (off-label use) was safe and effective in patients with DMO, nvAMD and MO following RVO [10–18]. Chhablani et al have reported that the 2mg dose of intravitreal ziv-aflibercept (IVZ) was safe and effective at 4 weeks after single injection [19]. A recent retrospective study (Singh et al, 2018) reported on the safety of IVZ in a large number participants [20].

There is no data on the safety and efficacy of IVZ in sub-Saharan Africa including Ghana to date, except for a few inclusions in the recent retrospective study of Singh et al (2018) [20]. This study evaluated the safety of 2 doses of ziv-aflibercept administered intravitreal injection in a randomised trial in Ghanaian patients with choroido-retinal vascular diseases.

## Materials and methods

This prospective randomized double-blind intervention study was conducted at the Eye Centre of Korle-Bu Teaching Hospital (KBTH) from December 2017 to March 2018. The study protocol received approval from Institutional Review Board of KBTH and Food and Drugs Authority (FDA) of Ghana (**FDA/CT/174)** and was registered at the Pan African Clinical Trial Registry (www.pactr.org) database (**PACTR201701001940111**). The study adhered to the Ghana Data Protection Act, and the tenets of declaration of Helsinki on human subjects. The study was monitored by a Data and Safety Monitoring Board, and the FDA of Ghana. Details of our study protocol have been deposited in protocols.io, dx.doi.org/10.17504/protocols.io.2dwga7e.

### Recruitment and eligibility

Consecutive patients with clinical diagnosis of DMO, nvAMD and MO following RVO were recruited into the study after obtaining written informed consent. Patients were eligible for the study if they met the following criteria: age 18 years or older, diagnostic criteria for DM, RVO and active nvAMD, treatment naïve patients, understand and willing to sign consent form, ability to comply with clinic visits, centre-involving MO in patients with diabetes mellitus and RVO with retinal thickness >300um using SD- OCT and, best corrected visual acuity (BCVA) of 6/12 (Snellen) (LogMAR 0.3) or worse. Exclusion criteria were glaucoma or IOP>21mmHg, intraocular surgery within 3 months in the study eye, history of uveitis, pregnancy or breastfeeding mothers, renal failure on dialysis or previous kidney transplant, allergy to active drug or excipients, cardiovascular events such as myocardial infarction or cerebrovascular accident, eye infections such as blepharitis, dacryocystitis, conjunctivitis or keratitis, and myopia ≥-6.0 Dioptres.

Medical history was taken from eligible patients including age, sex, history of hypertension, DM, hyperlipidaemia, cigarette smoking, current medications, duration of eye symptoms, diagnosis and any previous treatments. Comprehensive ocular examinations including: BCVA measurement using a standardised Early Treatment Diabetic Retinopathy Study (ETDRS) visual acuity chart (Precision Vision, La Salle, Illinois, USA) and recorded as logarithm of minimum angle of resolution (LogMAR), intraocular pressure (IOP) measured using Goldmann applanation tonometer, slit lamp biomicroscopy (Haag Strait model 900) examination of the anterior segment, and posterior segment examination with the aid of Volk 90D or 78D lenses. If both eyes were eligible, only the eye with the worse BCVA was recruited into the study. The other eye received standard care at a different visit. All patients had colour fundus photography (CFP) (Zeiss 450 Fundus Camera, Zeiss Inc. Jena, Germany), fundus fluorescein angiography (FFA) (Zeiss 450 Fundus Camera, Zeiss Inc. Jena, Germany) and spectral domain optical coherence tomography (SD-OCT) (Topcon 2000, Tokyo, Japan) at baseline. Systemic arterial blood pressure, fasting lipids and fasting blood sugar were also measured at baseline. The sphygmomanometer (Blanket MK-3, Accoson, England), SD-OCT and fundus camera were calibrated by Ghana Standard Authority prior to the commencement of the study.

#### Randomization

Eligible patients were randomly assigned to either 1.25mg (Group 1) or 2mg (Group 2) ziv-aflibercept using simple random sampling by a physician independent of the masked study investigators and the assigned dose of ziv-aflibercept was concealed to both patients and the masked trial (examining) ophthalmologists. Each patient received identical treatments at all treatment visits as per the original randomisation.

### Preparation of intravitreal ziv-aflibercept

The vial containing 100mg/4ml of ziv-aflibercept was punctured once under the laminar air flow system at the pharmacy manufacturing unit of KBTH and withdrawn using 5μ microfilter in 0.15ml aliquots into 1 ml syringes, labelled and each syringe kept in separate sterile plastic pouch (***Eye Drape plus***, ***Aurolab*, *India***) and were immediately stored at 4 degrees Celsius. Two (2) syringes containing the withdrawn samples were cultured on chocolate agar. Negative culture report was received before the remaining samples were released for injection and were used within 2 weeks from the date of preparation.

#### Intravitreal injection

This was performed by a certified physician who was independent of the masked clinical investigators. Standard precautions relating to intravitreal injections were observed. Intravitreal injections were given in the operating theatre using a sterile technique. Topical anaesthetic agent proparacaine and 5% povidone iodine were instilled into the conjunctival cul-de sac and periocular skin, eyelids and lashes cleaned using 10% povidone iodine. The eye was draped, 5% povidone instilled and the injection given in the inferotemporal quadrant using half inch 30-gauge needle into the mid vitreous cavity at 4 mm or 3.5mm posterior to the limbus in phakic and pseudophakic eyes, respectively and 5% povidone iodine was further instilled at the end of the procedure. Hand motion vision was checked and confirmed to be present. No topical antibiotics were given prior to, during or after each injection. IOP was measured 30 minutes post injection using Goldmann applanation tonometer. The intravitreal injections were repeated at 4 weeks and 8 weeks.

### Follow-up examinations

**Fig 1:**
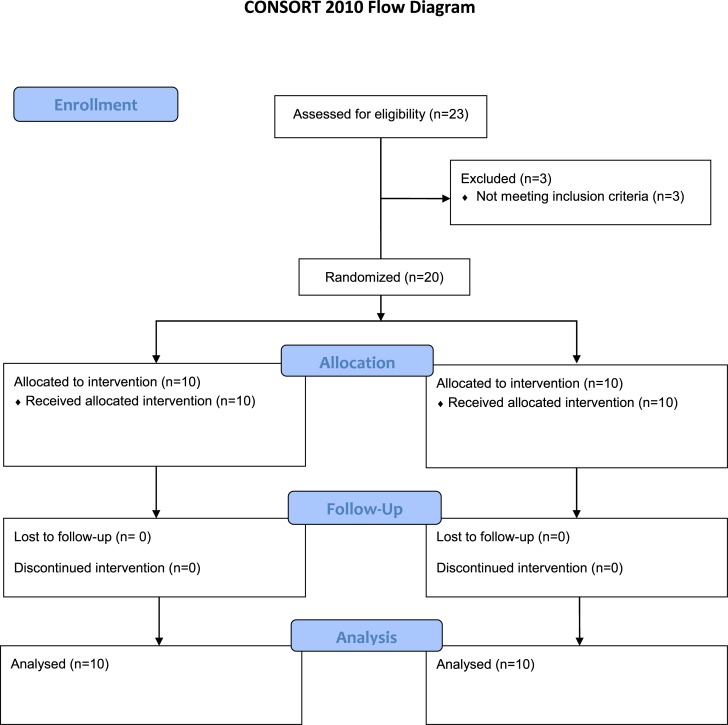
Flow of participant through the study.

Occurrence of ocular and systemic adverse events were assessed, and BCVA, IOP and slit lamp biomicroscope examination of the anterior and posterior segment were done on day 1 and 7 post initiation injection, and at 4 weeks, 8 weeks and 12 weeks. SD-OCT and fundus photography were done at all visits with the exception of day 1 post-injection. Fasting blood sugar and lipids were repeated on day 1 and 7 post-injection. The flow of participants through the study is shown in Fig 1.

### Image grading/analysis

Automated read outs from OCT scans (including retinal thickness) were recorded by examining masked local investigators. Anonymised CFP, FFA and SD-OCT were submitted electronically analysed by a remote masked independent investigator who was a trained retinal specialist (WMA) with expertise in retinal image grading. Any disparity in grading, as necessary, were to be adjudicated by a panel of 3 investigators (WMA/IZB/KAA).

### Outcome measures

The primary outcome measure in this study was safety of IVZ at 4 weeks. Ocular toxicity was assessed based on the number of ocular adverse events such as blurred vision (mild- loss of 0.1 LogMAR, moderate- loss of 0.2 LogMAR and severe—≥ 0.3 LogMAR), eye pain, raised intraocular pressure (>21mmHg), subconjunctival haemorrhage, conjunctival hyperemia, corneal abrasions, cataract, intraocular inflammation and endophthalmitis, retinal tears and retinal detachment, and deterioration of SD-OCT parameters including CSFT.

Intraocular inflammation/endophthalmitis was assessed using the standardised uveitis nomenclature (SUN) working group classification [21]. The definitive proof of endophthalmitis was dependent on vitreous biopsy and microbiological evaluation (microscopy, culture). Eye pain was assessed using the eye sensation scale [22].

Systemic adverse events were assessed based on systemic evaluation for the presence of hypertension, fever, gastrointestinal (GI) disorders, infections, neurologic disorder, Antiplatelet Trialists’ Collaboration (APTC) events including non-fatal myocardial infarctions, congestive heart failure, hospitalization and deaths, using predesigned questionnaire. Confirmation of systemic adverse event was done by the study physician.

Secondary outcome measures were occurrence of ocular and systemic adverse events at 12 weeks, IOP>25mmHg or increased IOP > 10mmHg from baseline, change in BCVA (LogMAR), CSFT and SD-OCT at 4 and 12 weeks from baseline.

The severity of ocular and systemic adverse events was determined using a toxicity grading scale and were categorised into mild, moderate, severe, potentially life threatening and death (S1 Table).

### Study monitoring

Monitoring for this study was done by a 3-member data and safety monitoring board and the Ghana FDA, to ensure that the rights and well-being of the enrolled patients were protected, that the reported trial data were accurate, complete, and verifiable, and that the conduct of the trial was compliant with Good Clinical Practice and with applicable Ghana FDA requirements.

### Statistical analysis

All statistical analyses for this study was done using STATA 13 (Statacorp, Texas, USA). The frequencies of ocular and systemic adverse events and serious adverse events were computed. Continuous variables were presented as mean and standard deviation (SD). Pre- and post-injection changes in BCVA, IOP, and CSFT were compared using paired t-test and categorical variables were compared using Fisher’s exact test or chi-square. Repeated-Measures ANOVA with nesting test was used to compare variations in continuous variables by IVZ dose, over time as well as interaction between IVZ dose and over time at times 0, 30 minutes post initiation of injection, day 1 and 7, and 4, 8 and 12 weeks. A p value<0.05 was considered statistically significant.

A standard study size of 8 eyes per dose has a 95% probability of detecting adverse events at a true rate of ≥32% at the specific dose. As such, if a specific particular adverse event is not observed in the 8 participants, there is a 95% confidence that the rate for the particular event at the specified dose is <32%. A sample size of 10 per group was chosen to allow for any potential drop out at study completion. All randomised subjects who received any study treatment and had at least 1 post-baseline BCVA in the study eye were included in full analysis set.

## Results

A total of 20 eyes (10 in group 1) of 20 treatment naïve patients who received IVZ and followed up for 12 weeks were included in this study. Their mean age (±Standard deviation) was 63.2± 7.37 years, and included 11 females. The main presenting complaint were blurred vison (18 patients) and metamorphopsia (2 patients) and the median duration of symptoms was 90 days (interquartile range, 60–112.5). The following systemic co-morbidities were observed: hypertension (17), diabetes mellitus (11), hyperlipidemia (2) and sickle cell disease genotype SS (1). Five patients consumed less than eight units of alcohol per week, and none smoked cigarette previously or currently. Eighteen eyes were phakic and 2 pseudophakic. The diagnosis included DMO (6), active nvAMD (7) and MO associated with RVO (7). The baseline clinical characteristics of the 2 groups were similar (Table 1).

**Table 1:** Baseline characteristics of 20 eyes on treatment with intravitreal ziv-aflibercept.

Parameter	IVZ 1.25mg (n = 10)	IVZ 2mg (n = 10)	P value
**Age in complete years:** Mean ± SD	63.0 ±6.7	63.4 ±8.4	0.91
**Study eye** right/left	5/5 (100%)	4/6 (100%)	1.00 ^®^
**Sex**: male/female	4/6 (100%)	5/5 (100%)	1.00^®^
**Lens status:** phakia/pseudophakia	9/1 (100%)	9/1 (100%)	1.00 ^®^
**PVD:** yes/no	1/9 (100%)	1/9 (100%)	1.00 ^®^
**Main presenting complaint**			
Blurred vision	8 (80%)	10 (100%)	0.47 ^®^
metamorphopsia	2 (20%)	0	
**Duration of symptoms:** Mean ± SD	81.0±42.5	119.8±84.8	0.21
**Diagnosis (number)**			0.50^™^
Diabetic macular oedema	3 (30%)	2 (20%)	
Neovascular AMD	3 (30%)	4 (40%)	
CME secondary to RVO	4 (40%)	4 (40%)	
**Systemic co-morbidities, yes/no**			
Diabetes mellitus	5/5 (100%)	4/6 (100%)	0.30^®^
Hypertension	8/2 (100%)	9/1 (100%)	1.00^®^
Hyperlipidemia	2/8 (100%)	1/9 (100%)	1.00 ^®^
Sickle cell	1/9 (100%)	0/10 (100%)	0.45^®^
**Blood pressure, mmHg**			
**Systolic**: Mean ± SD	144±17.1	142.2±22.4	0.84
**Diastolic**: Mean ± SD	83.5 ±6.7	81.6 ±11.8	0.66
**Fasting lipids (LDL-CHOL):** Mean ± SD	3.5 ±0.82	3.11 ±0.75	0.28
**Fasting blood sugar, mmol/l:** Mean ± SD	7.2 ±4.2	6.5 ±1.9	0.64
**Visual acuity, LogMAR:** Mean ± SD	1.0 ±0.6	0.85 ±0.6	0.49
**Central subfield fovea thickness(um):** Mean ± SD	382.2 ±128.4	429.6±153.8	0.46
**central fovea thickness:** Mean ± SD	435.6±201.8	386.2±153.5	0.54
**Intraocular pressure:** Mean ± SD	16.1±3.3	15.2±2.9	0.52

^™^ = chi square,

^®^ = Fischer’s exact test, AMD = age related macular degeneration, CME = cystoid macular edema, LDL-CHOL = low density lipoprotein cholesterol, LogMAR = logarithm of minimum angle of resolution, n = number, SD = standard deviation.

### Adverse events

Three patients (2 in group 1) reported eye pain of grade 2 (mild) severity on day 1 visit post injection. The pain resolved without treatment. No incidence of eye pain was reported in subsequent visits. Subconjunctival haemorrhage (1 eye in group 1), raised IOP >21mmHg at 30 minutes post-injection (5 eyes [50%] in group 2, 1 eye [20%] in group 1) were observed. IOP rise was graded as mild (grade 1) in all eyes because no eye had IOP rise >10mmHg from baseline. No treatment was given for the raised IOP and reverted to normal by day 1 after the initial injection. No eye had IOP >21mmHg on subsequent visits (Table 2).

**Table 2:** Ocular and systemic adverse events by treatment group.

Adverse event	Group 1, IVZ 1.25mg			Group 2, IVZ 2mg		
	Number of eyes = 10			Number of eyes = 10		
	Mild	Moderate	severe	Mild	moderate	Severe
	**Ocular**					
Blurred vision	0	0	0	0	0	0
Pain	2 (20%)	0	0	1(10%)		
Subconjunctival hemorrhage	1 (10%)	0	0	0	0	
Cornea abrasions	0	0	0	0	0	0
IOP>21mmHg¥	2 (20%)	0	0	5	0	0
Ocular Inflammation	0	0	0	0	0	0
Endophthalmitis	0	0	0	0	0	0
Retinal breaks/detachment	0	0	0	0	0	0
	**Systemic**					
Hypertension, hyperglycemia, chest pain, CCF, Infections, APTC-EVENTS, Death	0	0	0	0	0	0

Severity of adverse events is based on toxicity (adverse events) grading scale (S1 Table).

^¥^ = IOP>21mmHg was detected 30 minutes post injection only. APTC- antiplatelet trialists’ collaboration events (non-fatal myocardial infarctions, non-fatal strokes, or vascular deaths), CCF = congestive cardiac failure, IOP = intraocular pressure.

**Fig 2:**
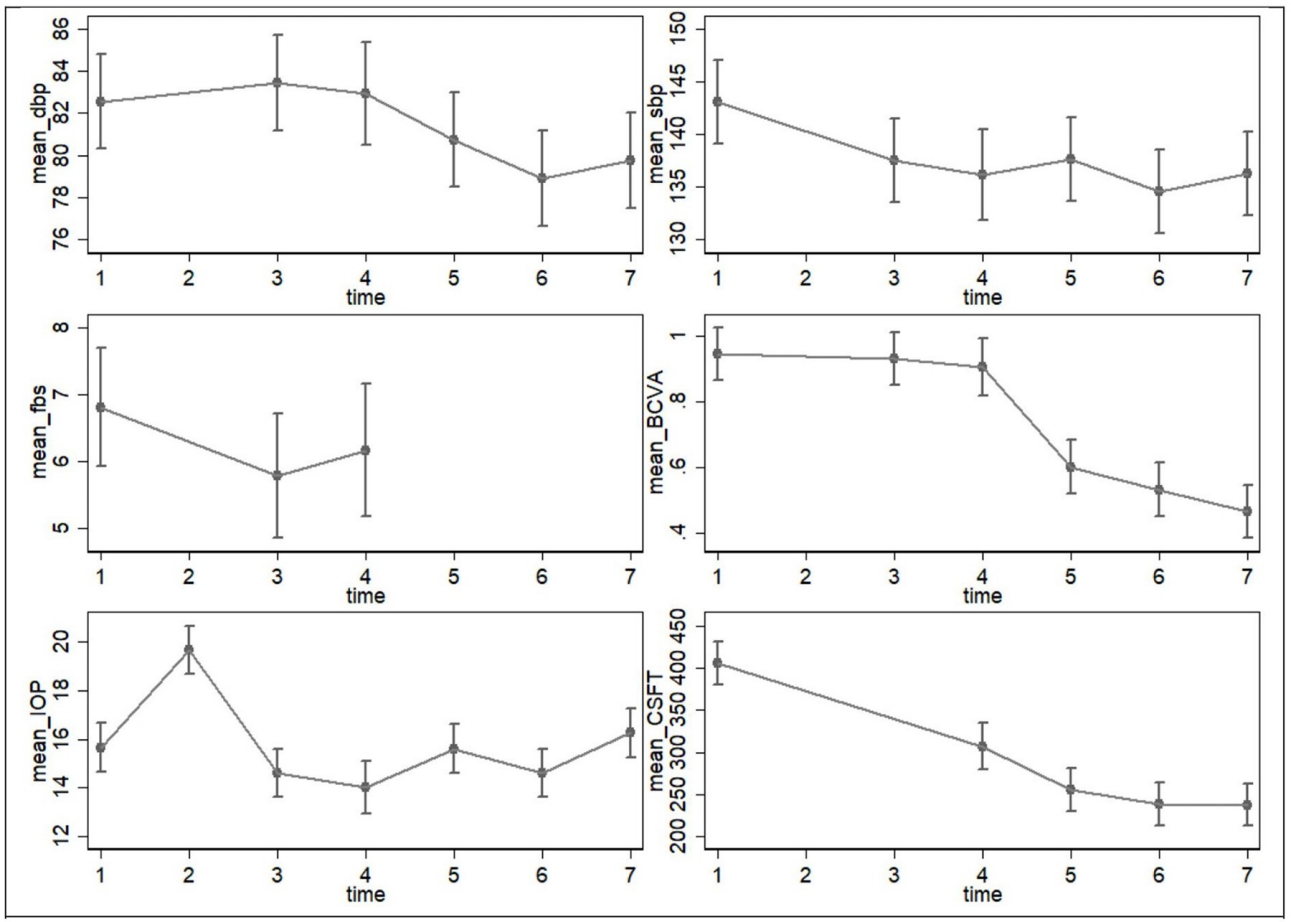
Mean change in clinical parameters over time with 2 standard error margin. 1 = baseline, 2 = 30 minutes post-injection, 3 = day 1, 4 = day 7, 5 = 4 weeks, 6 = 8 weeks, 7 = 12 weeks, BCVA = best corrected visual acuity, CSFT = central sub-field fovea thickness, dbp = diastolic blood pressure, fbs = fasting blood sugar, IOP = intraocular pressure, sbp = systolic blood pressure.

Variation in average IOP (± SD) was statistically significant over time (p<0.01); however, there was no significant variation between the IVZ dose and interaction (IVZ dose and time). The overall average IOP (± SD) for the whole study period was 15.8 ± 3.7. Although there was generally a significant reduction in average IOP over the study period, the averages fluctuated over the study period (Table 3 and Fig 2).

**Table 3:** Comparison of changes in continuous variables between IVZ dose at baseline and up to 12 weeks visit using Repeated-Measures ANOVA with nesting.

Variable	IVZ 1.25mg	IVZ 2mg	Overall	P-value*	P-value**	P-value***
	Mean ± SD	Mean ± SD	Mean ± SD			
**Systolic BP, mmHg**	140.1 ± 16.3	135.1 ± 17.3	137.6 ± 16.9			
Baseline	144 ± 17.1	142.2 ± 22.4	143.1 ± 19.5	0.50	0.08	0.45
Day 1	140.1 ± 17.7	135 ± 17.8	137.6 ± 17.4			
Day 7	142.8 ± 14.4	128.8 ± 10.9	136.2 ± 14.4			
4weeks	141.8 ± 17.7	133.5 ± 14.5	137.7 ± 16.3			
8weeks	136.1 ± 17.8	133.1 ± 15.6	134.6 ± 16.3			
12weeks	136 ± 14.9	136.5 ± 20.6	136.3 ± 17.5			
**Diastolic BP, mmHg**	83.2 ± 6.7	79.5 ± 11.3	81.4 ± 9.7	0.38	**0.03**	0.93
Baseline	83.5 ± 6.7	81.6 ± 11.8	82.6 ± 9.4			
Day 1	84.9 ± 6.7	82 ± 12.3	83.5 ± 9.7			
Day 7	85.6 ± 4.6	80 ± 12	82.9 ± 9			
4weeks	82.5 ± 7.2	79 ± 13.1	80.8 ± 10.4			
8weeks	81.5 ± 8.8	76.3 ± 11	78.9 ± 10.1			
12weeks	81.5 ± 5.8	78 ± 12.3	79.8 ± 9.5			
**Fasting blood sugar**	6.1 ± 2.7	6.4 ± 2.0	6.3 ± 2.4	0.75	0.22	0.34
Baseline	7.2 ± 4.2	6.5 ± 1.9	6.8 ± 3.2			
Day 1	5.5 ± 1	6.1 ± 1.4	5.8 ± 1.2			
Day 7	5.7 ± 1	6.7 ± 2.7	6.2 ± 2			
**Fasting Lipids Total Cholesterol**	5.4 ± 0.8	5.0 ± 0.9	5.2 ± 0.9	0.39	0.72	0.46
Baseline	5.5 ± 1	5.1 ± 1	5.3 ± 1			
Day 1	5.5 ± 0.8	5 ± 0.7	5.3 ± 0.8			
Day 7	5.1 ± 0.5	4.9 ± 1.2	5 ± 0.9			
**Fasting lipids LDL**	3.4 ± 0.8	2.9 ± 0.7	3.1 ± 0.7	0.35	0.14	0.72
Baseline	3.5 ± 0.8	3.1 ± 0.8	3.3 ± 0.8			
Day 7	3.1 ± 0.6	2.7 ± 0.5	2.9 ± 0.5			
**BCVA, LogMAR**	0.8 ± 0.5	0.7 ± 0.5	0.7 ± 0.5	0.46	**<0.01**	0.79
Baseline	1 ± 0.6	0.8 ± 0.6	0.9 ± 0.6			
Day 1	1 ± 0.6	0.8 ± 0.6	0.9 ± 0.6			
Day 7	0.9 ± 0.5	0.9 ± 0.5	0.9 ± 0.5			
4weeks	0.7 ± 0.4	0.5 ± 0.3	0.6 ± 0.4			
8weeks	0.6 ± 0.3	0.5 ± 0.3	0.5 ± 0.3			
12weeks	0.5 ± 0.3	0.4 ± 0.3	0.5 ± 0.3			
**IOP, mmHg**	15.2 ± 3.4	16.4 ± 3.9	15.8 ± 3.7	0.29	**<0.01**	0.85
Baseline	15.2 ± 2.9	16.1 ± 3.3	15.7 ± 3.1			
30 minutes	18.5 ± 3	20.8 ± 4.6	19.7 ± 4			
Day 1	14 ± 3.5	15.2 ± 3.1	14.6 ± 3.3			
Day 7	13.7 ± 2.8	14.4 ± 3.4	14 ± 3			
4weeks	15.2 ± 3.2	16 ± 3.7	15.6 ± 3.4			
8weeks	13.6 ± 2.2	15.6 ± 3.4	14.6 ± 2.9			
12weeks	15.9 ± 3.9	16.6 ± 3	16.3 ± 3.4			
**CSFT, um**	263.3 ± 106.4	314 ± 118.1	288.4 ± 114.6	0.16	**<0.01**	0.91
Baseline	382.2 ± 128.4	429.6 ± 153.8	405.9 ± 140			
Day 7	284.7 ± 88.5	331.5 ± 84.8	306.7 ± 87.4			
4weeks	222.9 ± 56.9	288.4 ± 79.1	255.7 ± 75			
8weeks	213.3 ± 69	263.8 ± 77.5	238.6 ± 76			
12weeks	215.7 ± 75.5	260.2 ± 97.5	238 ± 87.9			
**CFT, um**	248.6 ± 124.2	302.8 ± 147.1	275.4 ± 138	0.19	**<0.01**	0.62
Baseline	387.3 ± 156	429.8 ± 193.3	408.6 ± 172.4			
Day 7	264.7 ± 116.2	346.1 ± 122.6	303 ± 122.8			
4weeks	202.9 ± 69.7	271 ± 97.2	237 ± 89.5			
8weeks	199 ± 77.1	234.7 ± 96.3	216.9 ± 86.8			
12weeks	190.5 ± 68.7	240.8 ± 122.8	215.7 ± 100.2			

*P-value: P-value from ANOVA test for comparing means between drug dose,

**P-value: p-value from Huynh-Feldt epsilon for comparison of means over time period,

***P-value: p-values from Huynh-Feldt epsilon for comparison of means over drug dose and time period drug (interaction). BCVA = best corrected visual acuity, BP blood pressure, CFT = central fovea thickness, CSFT = central subfield fovea thickness, FBS = fasting blood sugar, IOP = intraocular pressure, LogMAR = logarithm of minimum angle of resolution, LDL = low density lipoprotein, CHOL = cholesterol, mmol/L = millimole per litre, SD = standard deviation.

No incidence of blurred vision, intraocular inflammation, cataract or endophthalmitis after the injection were observed in any study eyes throughout the study. No systemic adverse events were recorded. There was no significant difference in the fasting blood sugar, fasting lipids (total cholesterol and LDL cholesterol), blood pressure (systolic and diastolic) at any visit compared to baseline, and between the 2 groups (Table 3).

### Visual outcome

The average BCVA (LogMAR) level for the study period was 0.7 ± 0.5. The mean BCVA (LogMAR) (±SD) at baseline, day 1 and 7 and at 4, 8 and 12 weeks were 0.9 ± 0.6, 0.9±0.6, 0.9±0.5, 0.6±0.4, 0.5±0.3, and 0.5±0.3. The Repeated-Measures ANOVA with nesting test showed that the mean BCVA (LogMAR) did not vary significantly between the two IVZ doses but varied significantly across the study time period (P<0.01). Over the study period, average BCVA (LogMAR) improved marginally from baseline to Day 7 after which it improved drastically as shown in Fig 2. Interaction between IVZ dose and study time period was not statistically significant (Table 3). No eye had worsening of BCVA compared to baseline at any visit.

### Anatomic outcome

The overall average CSFT (± SD) for the whole study period was 288.4 ± 114.6 μm. Mean CSFT (± SD) at baseline, day 7, 4, 8 and 12 weeks were 405.9 ± 140μm, 306.7 ± 87.4μm, 255.7 ± 75μm, 238.6 ± 76μm and 238 ± 87.9μm, respectively. Generally, there was significant reduction in the average CSFT (± SD) over the study period from 405.9 ± 140μm at baseline to 238 ± 87.9μm after 12 weeks (p<0.0001) however there was no significant variation between the IVZ dose and interaction (IVZ dose and time) (Table 3 and Fig 2). No disparities occurred in image grading/analysis that required adjudication.

**Fig 3:**
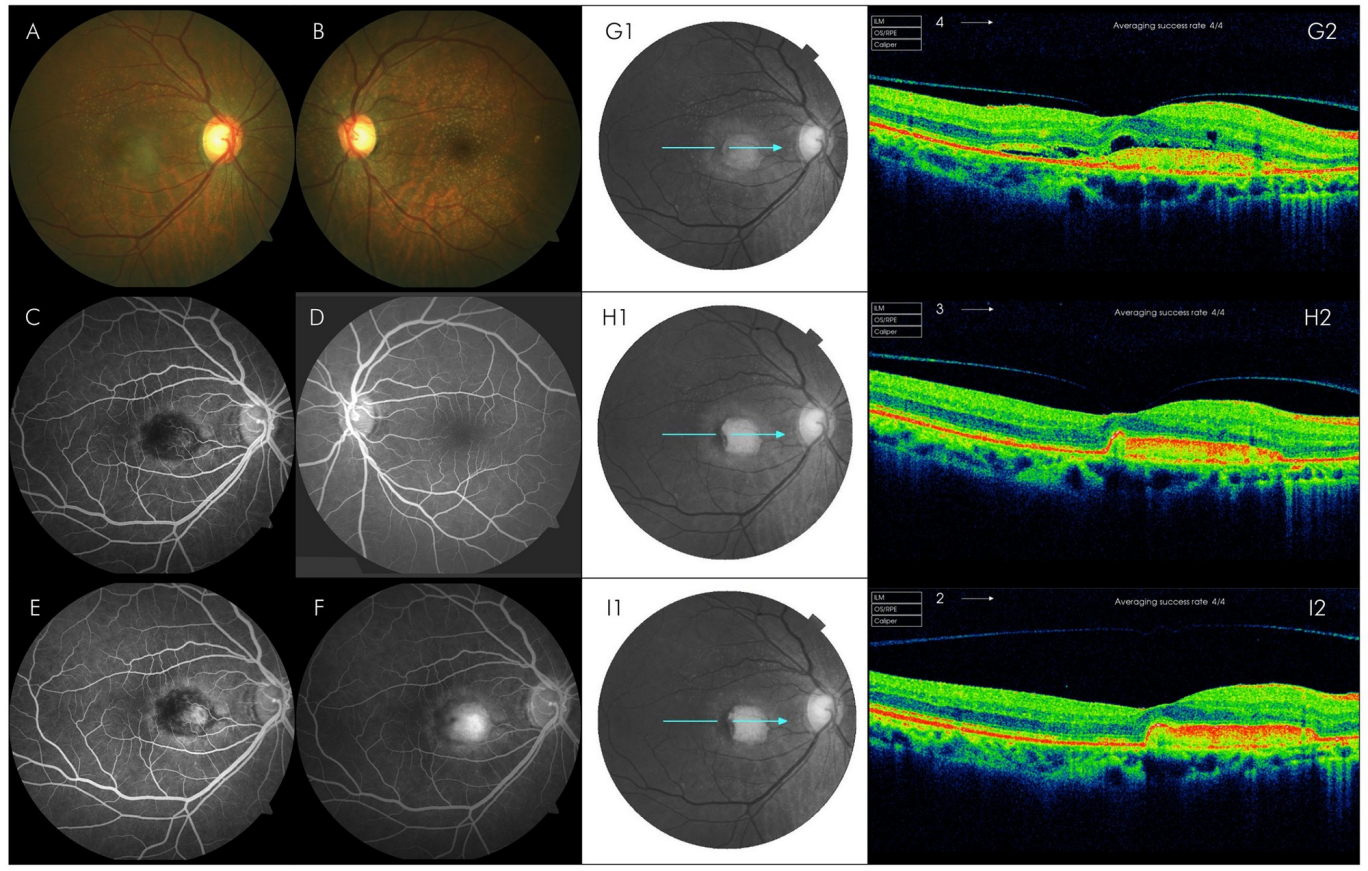
Fundus photograph (CFP), fluorescein angiogram (FFA) and spectral domain optical coherence tomography (SD-OCT) of a 56-year female presenting with metamorphosia in the right eye of 2 months’ duration. CFP (A and B) shows drusen and reticular pseudodrusen (RPD) in both eyes with subfoveal CNV in the right eye confirmed with FFA (C-F). SD-OCT showed vitreomacular adhesion (VMA), intraretinal (IRF) and subretinal fluid (IRF) and retinal pigment epithelial detachment (PED) at baseline(G), with resolution of IRF and SRF at 4 (H) and 12 (weeks) and separation of the VMA at 12 weeks (I). Visual acuity at baseline of 0.4 LogMAR improved to 0.24 LogMAR and 0.2 LogMAR at 4 and 12 weeks, respectively.

Of the 7 eyes with nvAMD, there was significant reduction in the mean CSFT from 281.6 ±105μm at baseline to 197±46μm (p = 0.03) and 188.4 ±86μm (p = 0.02) at 4 and 12 weeks respectively. Similarly, of the 7 eyes with MO associated with RVO, there was significant reduction in the mean CSFT to 255.9 ±56 μm (p<0.01) and 225.1 ±23 μm (p<0.01) at 4 and 12 weeks respectively compared to 488.4 ±124 μm at baseline. Of the 6 eyes with DMO, there was significant reduction in the mean CSFT to 323.8 ±67 μm and 310.7 ±99 μm at 4 and 12 weeks respectively compared to 454.7 ±93 μm at baseline. Images of a case of nvAMD in the right eye is shown in Fig 3.

## Discussion

In this prospective randomized double-blind intervention study, no difference in ocular and systemic adverse events, and the anatomic and visual outcome were found between the 1.25mg and 2mg doses of IVZ at 4 and 12 weeks in treatment naïve eyes of Ghanaian patients with DMO, nvAMD and MO following RVO. Specifically, no significant difference was observed in eyes receiving the 2mg compared to 1.25mg of IVZ as would be expected from the larger volume administered with the higher drug dose.

Ziv-aflibercept and aflibercept are identical fusion proteins comprising of the Fc portion of human immunoglobin IgG1 and extracellular matrix domains of VEGF receptors 1 and 2 [15]. They act as decoy receptors by binding to circulating VEGF- A and B and placental growth factor. Ziv-aflibercept is an 115kDa molecule manufactured from Chinese hamster ovary cells [15]. The molecular structure of ziv-aflibercept is identical to aflibercept. However, the 2 drugs are different because they undergo different purification processes, and whilst aflibercept is iso-osmotic (300mosm/kg) ziv-aflibercept is hyperosmotic (1000mosm/kg) due to addition of higher concentration of sucrose [15, 23]. Ziv-aflibercept is packaged as 25mg/ml of ziv-aflibercept in polysorbate 20 (0.1%), sodium citrate (5 mM), sodium phosphate (5 mM) and sucrose (20%), in water for Injection USP, at a pH of 6.2 and aflibercept as 40mg/ml aflibercept in 10 mM sodium phosphate, 40 mM sodium chloride, 0.03% polysorbate 20, and 5% sucrose, pH 6.2 [15]. Aflibercept has been approved by the USA FDA for ocular use whilst ziv-aflibercept was approved for the treatment of metastatic colorectal cancers and other cancers [7]. There had been earlier concerns about the intravitreal administration of ziv-aflibercept due to hyper-osmolality, but the potential retinal toxicity from its hyper-osmolality has been refuted by several studies [12, 15, 18, 24–30]. Malik et al has shown that the use of 1.25mg and 2mg doses of ziv-aflibercept did not affect the viability of human retinal pigment epithelial cells in vitro although there was a mild reduction in mitochondrial membrane potential with the 2mg dose [31]. Similarly, de Oliveira Dias (2015) reported that ziv-aflibercept was safe in the rabbit eye when given intravitreally in doses up to 25mg/ml [32]. Mansour et al (2015) and Chhablani et al (2016) reported that the intravitreal administration of 1.25mg of ziv-aflibercept did not show ocular toxicity at 4 weeks in eyes with DMO and nvAMD [12, 15]. In the present study, 3 patients reported of mild pain on day 1 and 6 had elevation of IOP at 30 minutes post-injection. The recommended dose of intravitreal aflibercept is 2mg/0.05ml. A higher volume of ziv-aflibercept (2mg/0.08ml) is required to achieve same dose as aflibercept and can potentially lead to a rise in IOP. Chhablani et al (2017) did not observe significantly raised IOP (>21mmHg) at 30 minutes post-injection of 2mg IVZ [19]. However, 4 of the 21 eyes in their study had anterior chamber paracentesis because digital examination suggested immediate IOP increase [19]. In our study, we observe increased IOP>21mmHg in 5 eyes who received 2mg IVZ; however, none of the eyes had IOP increase beyond 10mmHg from baseline and the elevation of IOP reverted to normal range without treatment. Intravitreal injections of pegaptanib (Macugen, Pfizer), the first anti-VEGF agent licensed for intraocular injection was approved to be delivered at a dose of 0.3mg in 0.09ml of volume [33]. Although there was transient elevation of IOP, this did not translate into serious adverse events [34].

Although we did not observe significant difference in the visual and anatomic outcome between the 1.25mg and 2mg dose of IVZ at 4 weeks and 12 weeks, the number of eyes in our study was small but compatible with our primary focus to determine the safety of the 2 different doses of IVZ in a Ghanaian population with DMO, nvAMD and RVO, but not powered for efficacy. This corroborates the results of the three armed double blind study (which included 123 eyes) of Baghi et al which did not observe significant difference in the visual and anatomic outcome between eyes with DMO who received 1.25mg (42 eyes) versus 2.5mg (42 eyes) at 12 weeks(28) or study extension to 1 year [35]. Several short-term and a few long term studies from other populations elsewhere have shown that the 1.25mg dose of IVZ was safe and effective when administered to eyes with DMO, nvAMD and RVO [10–17]. The results are supported by the recent retrospective study of Singh et al (2018) [20].

The limitations of our study are the inclusion of small number of eyes with varied clinical diagnosis and short duration of follow-up. However, the primary focus of this study was to systematically evaluate the safety of the 2 different doses of IVZ in eyes with DMO, nvAMD and RVO, as a prelude to further studies on efficacy. The doses chosen were limited by the concentration of the commercially available ziv-aflibercept. As such a wider dose range was not possible as the drug volume delivered intravitreal would have resulted in potential under-dosing (e.g. at 0.02 mls for a lower dose), or too large a volume (e.g. 0.15mls for a higher dose). The efficacy and safety of IVZ in a Ghanaian population with retinal vascular diseases will need to be confirmed through further studies with larger numbers and a longer duration of follow-up. The other limitations of this study are that pharmacodynamic measurements and genetic testing were not perfromed.

## Conclusion

The 1.25mg and 2mg dose of ziv-aflibercept administered at 4-weekly intervals was found to be safe up to 12 weeks in the eyes of Ghanaian Africans with DMO, nvAMD, and MO secondary to RVO. The adverse events observed were mild and resolved without treatment. There was a tendency for improvement in BCVA at 12 weeks and reduction in CMT at 12 weeks. A randomized prospective study which includes a larger number of participants evaluated over a longer time period is required to verify our findings and establish the efficacy of IVZ in retinal diseases in the African.

## Supporting information

S1 Table Toxicity grading scale. (PDF)

S2 Table Clinical data of 20 eyes of 20 patients with retino-choroidal vascular diseases. (PDF)

S1 Protocol Safety of ziv-aflibercept Ghana. (PDF)

S1 Consort checklist (DOC)
