# Supplementary material for: Safety of intravitreal ziv-aflibercept in choroido-retinal vascular diseases: A randomised double-blind intervention study
Source: PLoS One. 2019 Oct 24;14(10):e0223944. doi: 10.1371/journal.pone.0223944 (PMC6812750; doi:10.1371/journal.pone.0223944)
Supplement: S1 Table — (PDF) [file pone.0223944.s001.pdf]

TOXICITY GRADING SCALE

GRADE	DEFINITIONS
1	Mild symptoms causing no or minimal interference with usual social & functional activities with intervention not indicated
2	Moderate symptoms causing greater than minimal interference with usual social & functional activities with intervention indicated
3	Severe symptoms causing inability to perform usual social & functional activities with intervention or hospitalization indicated
4	Potentially life-threatening symptoms causing inability to perform basic self-care functions with intervention indicated to prevent permanent impairment, persistent disability, or death

GRADE 5= death

Table for Grading the Severity of Adverse Events Associated with Intravitreal Ziv-Aflibercept Injection

PARAMETER	GRADE 1 Mild	GRADE 2 Moderate	GRADE 3 severe	GRADE 4 potentially life-threatening
Blurred Vision	<0.1 LogMAR	0.1-0.2 LogMAR	≥0.3 LogMAR	NA
Conjunctiva	Hemorrhage/ injection	NA	necrosis	NA
Sclera	NA	NA	necrosis	NA
cornea	Abrasion transient does not require treatment	Corneal abrasion requiring treatment	Persistent corneal abrasion despite treatment OR corneal edema	NA
Anterior chamber	Grade 1 cells and flare	Grade 2 cells and flare	Grade 3 or 4 flare and cells	NA
Vitreous	Grade 1 vitritis PVD	Grade 2 vitritis	Grade 3 or 4 vitritis Vitreous hemorrhage	NA
lens	NA	NA	Cataract of any type	NA
retina	NA	NA	Retinal tear, detachment or hemorrhage. Optic atrophy	NA
Endophthalmitis	NA	NA	Proven case of endophthalmitis(eye infection)	NA
pain	Grade 1 and 2	Grade 3	Grade 4 and 5	NA
Intraocular pressure	<10mmHg increase IOP from baseline	Between 10-15mmHg increase IOP from base line	>15mmHg increase IOP from baseline	NA
Blood pressure	140 to < 160 mmHg systolic OR 90 to < 100 mmHg diastolic	≥ 160 to < 180 mmHg systolic OR ≥ 100 to < 110 mmHg diastolic	≥ 180 mmHg systolic OR ≥ 110 mmHg diastolic	Life-threatening consequences in a participant not previously diagnosed with hypertension (e.g.,

				malignant hypertension) OR Hospitalization indicated
Temperature	38.0 to < 38.6°C or	≥ 38.6 to < 39.3°C	≥ 39.3 to < 40.0°C or	≥ 40.0°C
Fasting Blood sugar	Controlled without medication	Controlled with medication OR Modification of current medication regimen	Uncontrolled despite treatment modification OR Hospitalization for immediate glucose	Life-threatening consequences (e.g., ketoacidosis, hyperosmolar nonketotic coma
Fasting lipids	LDL cholesterol <129mg/dL	LDL cholesterol between 130 to 159mg/dL	LDL cholesterol between 160 to 189	190 mg/dL or more
Congestive heart failure	No symptoms AND Laboratory or cardiac imaging abnormalities	Symptoms with mild to moderate activity or exertion	Symptoms at rest or with minimal activity or exertion (e.g., hypoxemia) OR Intervention indicated (e.g., oxygen)	Life-threatening consequences OR Urgent intervention indicated (e.g., vasoactive medications, ventricular assist device
APTC- EVENTS	NA	NA	Transient ischemic attack, New symptoms with ischemia (stable angina) OR New testing consistent with ischemia	stroke with neurological deficit, Unstable angina OR Acute myocardial infarction, Pulmonary embolism
GIT disorders/ bleeding	Not requiring intervention other than iron supplement	Endoscopic intervention indicated	Transfusion indicated	Life-threatening consequences (e.g., hypotensive shock)
infections		Non-parenteral treatment indicated	IV treatment indicated	Life-threatening consequences (e.g., sepsis, tissue necrosis)
Neurologic disorder	Changes causing no or minimal interference with usual social & functional activities	Mild lethargy or somnolence causing greater than minimal interference with usual social & functional activities	Confusion, memory impairment, lethargy, or somnolence causing inability to perform usual social & functional activities	Delirium OR Obtundation OR Coma
Wound healing complications	Incisional separation of ≤25% of wound, no deeper than superficial fascia	Incisional separation >25% of wound with local care;	fascial disruption/dehiscence without evisceration; primary wound closure or revision by operative intervention	fascial disruption with evisceration; major reconstruction flap, grafting, resection, or amputation indicated

		asymptomatic hernia	indicated; hospitalization or hyperbaric oxygen indicated	

APTC- antiplatelet trialist's collaboration (EVENTS- non-fatal myocardial infarctions, non-fatal strokes, or vascular deaths). GIT: gastrointestinal tract. IOP: intraocular pressure. NA: not applicable.

Mortality: Neither the exudative retinal diseases nor their treatments are significantly associated with mortality. All deaths related to an adverse event are classified as grade 5. Any reported death shall be communicated to the DSMB within 24hrs and the FDA Ghana within 48hr

®Grading of severity of systemic adverse events modified from DAIDS AE Grading Table Version 2.0- November 2014.
