# Supplementary material for: Safety of intravitreal ziv-aflibercept in choroido-retinal vascular diseases: A randomised double-blind intervention study
Source: PLoS One. 2019 Oct 24;14(10):e0223944. doi: 10.1371/journal.pone.0223944 (PMC6812750; doi:10.1371/journal.pone.0223944)
Supplement: S2 Table — (PDF) [file pone.0223944.s002.pdf]

S2 Table 2a, 2b, 2c. Clinical data of 20 eyes of 20 patients with retino-choroidal vascular diseases.

Table 2a. Baseline demographic and clinical characteristics of study eyes

**Serial no.**	Eye	Age	Sex	Main Complaint	Symptoms (days)	Alcohol intake	Diabetes Mellitus	Hyper-Tension	Hyper-Lipidemia	Sickle cell Disease
**1**	LEFT	63	F	Blurred vision	180	No	Yes	yes	unknown	no
**2**	LEFT	62	F	Blurred vision	14	No	No	no	unknown	no
**3**	LEFT	63	M	Blurred vision	180	No	Yes	yes	unknown	no
**4**	RIGHT	56	F	metamorphopsia	60	No	No	no	unknown	yes, SS
**5**	LEFT	78	F	Blurred vision	60	No	No	yes	unknown	no
**6**	LEFT	60	F	Blurred vision	90	No	No	yes	unknown	no
**7**	RIGHT	78	M	metamorphopsia	30	yes	Yes	yes	no	no
**8**	RIGHT	54	F	Blurred vision	240	No	No	yes	unknown	no
**9**	RIGHT	53	M	Blurred vision	90	yes	Yes	yes	yes	no
**10**	RIGHT	69	M	Blurred vision	180	No	Yes	yes	unknown	no
**11**	LEFT	62	M	Blurred vision	90	yes	Yes	no	yes	no
**12**	LEFT	64	F	Blurred vision	90	No	No	yes	unknown	no
**13**	RIGHT	63	F	Blurred vision	14	No	Yes	yes	unknown	no
**14**	LEFT	55	F	Blurred vision	90	No	No	yes	unknown	no
**15**	RIGHT	59	F	Blurred vision	30	No	No	yes	unknown	no
**16**	LEFT	62	M	Blurred vision	90	No	No	yes	unknown	no
**17**	LEFT	61	M	Blurred vision	240	yes	No	yes	yes	no
**18**	RIGHT	77	M	Blurred vision	90	No	Yes	yes	unknown	no
**19**	LEFT	66	F	Blurred vision	60	No	No	yes	unknown	no
**20**	RIGHT	59	M	Blurred vision	90	yes	Yes	yes	unknown	no

Table 2b. Baseline clinical characteristics of study patients.

SERIAL NO.	systolic BP mmHg	Diastolic BP mmHg	blood sugar mmol/Dl	LDL-chol mmol/L	BCVA (logMAR)	IOP mmHg	Lens status	SD-OCT CENTRE 1mm	Diagnosis
1	130	60	7.7	3.6	0.48	18	Phakia	574	DME
2	140	80	5.1	2.4	2.3	13	Phakia	163	CNV
3	180	90	18.3	3.5	0.5	19	Phakia	319	DME
4	120	70	5	1.9	0.4	16	Phakia	284	CNV
5	130	90	5.3	3.6	1.5	18	Phakia	613	HRVO
6	140	80	4.7	3.2	1.02	16	Phakia	472	CNV
7	160	80	5.4	2.6	0.32	20	Phakia	198	PPCNV
8	130	70	5.4	3.2	0.5	16	Phakia	316	BRVO
9	180	90	7.4	4.4	0.48	12	Phakia	535	DME
10	130	80	7.2	4.4	0.78	15	Phakia	461	BRVO
11	110	80	10.2	3.2	0.5	10	Phakia	475	DME
12	124	72	6	3.6	1.8	20	Pseudophakia	226	CNV
13	140	90	4.7	3.5	0.48	15	Phakia	428	DME
14	130	90	9.6	3.9	1.3	14	Phakia	358	CNV
15	150	100	4.5	2.3	0.6	18	Phakia	400	CRVO
16	158	84	4.5	2.6	1.8	14	Phakia	594	HRVO
17	130	85	4.7	4.2	1.8	14	Phakia	404	HRVO
18	160	80	4.7	2.1	1.3	10	Pseudophakia	270	CNV
19	160	90	7.1	4.6	0.46	20	Phakia	631	CRVO
20	160	90	8.6	3.3	0.6	15	Phakia	397	DME

BP= blood pressure, BRVO= branch retinal vein occlusion, CNV= choroidal neovascularization, CRVO= central retinal vein occlusion, DME= diabetic macular edema, HRVO= hemiretinal vein occlusion,IOP= intraocular pressure, LDL-chol= low density lipoprotein cholesterol, SD-OCT= spectral domain optical coherence tomography. PPCNV= peripapillary choroidal neovascularization.

Table 2c: Clinical characteristics and adverse events of 20 eyes on day 1 and 7, and at 4, 8 and 12 wks.																				
Parameters	Serial number of patients																			
	1	2	3	4	5	6	7	8	9	10	11	12	13	14	15	16	17	18	19	20
IOP mmHg																				
30 min after injection	24	18	20	21	26	16	25	20	16	20	14	25	16	13	24	15	20	18	24	18
day 1	18	6	17	16	16	13	16	14	16	18	9	16	15	14	20	12	13	12	18	13
day 7	15	10	16	13	18	14	x	14	x	19	9	14	14	11	20	13	11	x	14	13
4 weeks	19	9	19	17	21	14	20	12	16	16	10	12	14	16	18	13	16	16	20	14
8 weeks	16	9	13	18	20	16	20	13	16	14	10	12	14	12	16	12	13	16	16	16
12 weeks	15	12	16	18	21	16	21	14	16	26	16	12	13	13	18	13	16	17	16	16
Systolic BP, mmHg																				
Day 1	120	127	184	110	140	130	140	130	170	140	120	120	130	130	140	150	130	150	150	140
Day 7	140	130	175	115	130	140	x	130	x	130	150	120	115	130	140	130	140	x	150	140
4 weeks	130	140	178	110	140	140	130	120	160	120	120	125	130	140	140	130	140	150	160	150
8 weeks	140	140	180	100	130	140	146	116	150	130	130	120	140	125	140	125	130	120	140	150
12 weeks	135	110	165	100	160	130	130	140	170	130	120	120	125	150	150	140	130	140	140	140
Diastolic BP, mmHg																				
Day 1	70	84	90	60	90	85	80	70	90	90	80	70	90	90	90	90	80	80	90	100
Day 7	80	80	90	60	90	90	x	80	x	90	70	70	80	90	90	90	85	x	85	90
4 weeks	70	70	95	50	100	80	80	80	90	80	80	75	80	90	80	80	80	80	90	85
8 weeks	65	80	85	60	90	90	78	60	90	90	80	60	80	90	80	80	80	80	80	80
12 weeks	70	70	90	50	90	80	80	80	90	80	80	70	80	90	80	80	85	80	80	90
Fasting blood sugar mmol/dL																				
Day 1	5	4.8	7	4.4	5.3	5.1	5.8	5.3	7.2	7.4	7.4	5.7	5.6	5.4	5.4	5.8	5.6	4.7	4.5	8.3
Day 7	9.4	5.6	6	4.5	5.4	5	x	5.4	x	7.8	6.2	5.6	5.3	5.4	4.9	5.1	4.7	x	6.1	12.3
LDL cholesterol																				

Day 1	2.9	2.3	3.4	1.8	2.9	2.7	3.1	3	3.1	3.1	2.6	3.7	3.8	3.8	2.6	3.1	4.1	3.2	4.4	2.4
Day 7	3.3	2.7	3.5	2.4	2.7	2.9	x	3	x	2.9	2.4	2.9	2.9	2.4	2.3	2.4	3.9	x	2.9	3.5
BCVA in logMAR																				
Day 1	0.48	2.3	0.5	0.4	1.5	1.0	0.32	0.5	0.48	0.78	0.5	1.8	0.48	1	0.6	1.8	1.8	1.3	0.46	0.6
Day 7	0.6	1.8	0.5	0.4	1.5	1.0	x	0.5	x	0.8	0.46	1.3	0.48	1	0.6	1.5	1.8	x	0.42	0.72
4 weeks	0.34	1.3	0.4	0.24	1	1	0.2	0.3	0.32	0.46	0.34	1	0.24	0.66	0.4	1	1.3	0.84	0.3	0.4
8 weeks	0.3	1.3	0.5	0.22	1	0.8	0.3	0.5	0.2	0.4	0.32	0.9	0.14	0.5	0.3	0.98	0.72	0.8	0.14	0.34
12 weeks	0.2	1.2	0.5	0.2	0.94	0.78	0.18	0.4	0.2	0.4	0.2	0.5	0.14	0.44	0.3	0.88	0.72	0.72	0.1	0.3
SD-OCT (centre 1mm) **um**																				
Day 7	354	132	275	267	437	430	x	286	x	282	374	206	280	399	297	443	260	x	218	274
4 weeks	330	151	280	197	298	285	198	248	450	282	339	206	267	198	261	328	161	144	213	277
8 weeks	373	98	231	203	280	343	173	232	413	263	255	179	255	192	262	252	173	137	209	248
12 weeks	403	90	260	194	256	363	172	209	465	238	251	178	266	196	211	253	199	126	210	219
Adverse events (grades)																				
Blurred vision	0	0	0	0	0	0	0	0	0	0	0	0	0	0	0	0	0	0	0	0
pain	0	0	0	2	0	0	2	0	0	0	0	0	0	0	0	0	2	0	0	0
conjunctiva	0	0	0	0	0	0	1	0	0	0	0	0	0	0	0	0	0	0	0	0
cornea	0	0	0	0	0	0	0	0	0	0	0	0	0	0	0	0	0	0	0	0
Anterior chamber	0	0	0	0	0	0	0	0	0	0	0	0	0	0	0	0	0	0	0	0
lens	0	0	0	0	0	0	0	0	0	0	0	0	0	0	0	0	0	0	0	0
vitreous	0	0	0	0	0	0	0	0	0	0	0	0	0	0	0	0	0	0	0	0
retina	0	0	0	0	0	0	0	0	0	0	0	0	0	0	0	0	0	0	0	0
endophthalmitis	0	0	0	0	0	0	0	0	0	0	0	0	0	0	0	0	0	0	0	0
Fever	0	0	0	0	0	0	0	0	0	0	0	0	0	0	0	0	0	0	0	0
Surg./medical procedure	0	0	0	0	0	0	0	0	0	0	0	0	0	0	0	0	0	0	0	0

Wound healing problem	0	0	0	0	0	0	0	0	0	0	0	0	0	0	0	0	0	0	0	0
GIT disorders/ bleeding	0	0	0	0	0	0	0	0	0	0	0	0	0	0	0	0	0	0	0	0
Infections	0	0	0	0	0	0	0	0	0	0	0	0	0	0	0	0	0	0	0	0
Neurologic disorder	0	0	0	0	0	0	0	0	0	0	0	0	0	0	0	0	0	0	0	0
Hospitalization	0	0	0	0	0	0	0	0	0	0	0	0	0	0	0	0	0	0	0	0
Congestive heart failure	0	0	0	0	0	0	0	0	0	0	0	0	0	0	0	0	0	0	0	0
APTC events	0	0	0	0	0	0	0	0	0	0	0	0	0	0	0	0	0	0	0	0
Death	0	0	0	0	0	0	0	0	0	0	0	0	0	0	0	0	0	0	0	0

X= missing data due to missed appointment, 0= no adverse event observed, 1= adverse event of mild severity (S1 table 1- toxicity grading scale). 2= pain of mild intensity (Eye sensation scale). APTC- Antiplatelet trialists' collaboration events, BP= blood pressure, GIT= gastrointestinal tract, IOP= intraocular pressure, SD-OCT= spectral domain optical coherence tomography, Surg=surgery.
