# Supplementary material for: Safety of intravitreal ziv-aflibercept in choroido-retinal vascular diseases: A randomised double-blind intervention study
Source: PLoS One. 2019 Oct 24;14(10):e0223944. doi: 10.1371/journal.pone.0223944 (PMC6812750; doi:10.1371/journal.pone.0223944)
Supplement: S1 Protocol — (PDF) [file pone.0223944.s003.pdf]

STUDY PROPOSAL

TITLE: Safety of intravitreal ziv-aflibercept in patients with diabetic macular edema, macular edema following retinal vein occlusion and neovascular age related macular degeneration in a Ghanaian population: A phase I randomized interventional study.

Short Title: Safety of ziv-aflibercept in retinal diseases in a Ghanaian population. A randomized interventional study.

Principal Investigator (PI).

[1, 2] Imoro Zeba Braimah (IZB). MBChB, FGCS, FWACS. Tel: 0206301353

E-mail: zebaimoro2000@yahoo.com, izbraimah@ug.edu.gh

Co-Investigators:

[1, 2] Akafo Stephen (AS), MBChB, FGCS, FWACS, FRCOphth. skakafo@chs.edu.gh, Tel: 0208170900

[1, 2] Kwesi Amissah-Arthur (KA), MBChB, FRCOphth. FGCS kwesi@amissaharthur.com, Tel: 027 6864343

[4]W. M. Amoaku (WMA), MBChB, FRCOphth. winfried.amoaku@nottingham.ac.uk Tel: +441158231014

[3]Ernest Kenu (EK), MBChB, MGCP, MPH, PhD. Ernest_kenu@yahoo.com 024459212

INSTITUTIONS

1. Department of surgery (Eye), School of Medicine and Dentistry, University of Ghana.
2. Eye Centre, Korle- Bu Teaching Hospital. Korle- Bu.
3. Department of Community Health, School of Public Health, University of Ghana
4. Academic Ophthalmology, DCN, Faculty of Medicine and Health Sciences, University of Nottingham, Nottingham.

SOURCE OF FUNDING: Book and Research allowance (PI), Eye Centre, and Korle-Bu Teaching Hospital.

LIST OF ABBREVIATIONS.

AEs	Adverse events
BCVA	Best corrected visual acuity
BRAVO	BRAnch Retinal Vein Occlusion: Evaluation of Efficacy and Safety
CATT	Comparison of Age-Related Macular Degeneration Treatment Trials
CRUISE	Central Retinal Vein OcclUsIon Study
CSFT	central subfield foveal thickness
DME	Diabetic macular edema
DRCR.net	Diabetic Retinopathy Clinical Research Network
EMA	European Medicines Agency
ETDRS	Early Treatment Diabetic Retinopathy Study
FDA	food and drug administration
IVAN	Inhibition of VEGF in Age-related choroidal Neovascularisation
logMAR	Logarithm of minimum angle of resolution
nvAMD	neovascular age related macular degeneration
RVO	retinal vein occlusion
SAE	Serious adverse event
SD-OCT	Spectral Domain Optical Coherence Tomography
SUN	Standardisation of uveitis nomenclature
USD	United States Dollars
VEGF	Vascular endothelial growth factor
VIEW	VEGF Trap-Eye: Investigation of Efficacy and Safety in Wet AMD
VISION	VEGF Inhibition Study In Ocular Neovascularization

Structured Abstract

Brief Background: Retinal vascular diseases are a significant cause of visual loss world-wide, including Ghana. Anti-vascular endothelial growth factor (anti-VEGF) such as aflibercept, ranibizumab and bevacizumab have become the standard of care for diabetic macular edema (DME), macular edema (ME) following retinal vein occlusions (RVO), and neovascular age related macular degeneration(nvAMD). Aflibercept has been approved by the United States of America (USA) Food and Drug Administration (FDA) for the treatment of DME, nvAMD, and ME following RVO. The recommended dose of intravitreal aflibercept is 2mg in 0.05ml administered monthly or initial monthly injections for the first 3 months followed by 2 monthly injections. Aflibercept is highly expensive (USD1850 per dose) and not available in many developing countries including Ghana. Ziv-aflibercept, a molecule structurally identical to aflibercept but differs due to its formulation with hyper-osmolality has been approved by the USA FDA for the treatment of metastatic colorectal cancers. Ziv-aflibercept, used off-label, has been found to be safe in patients with DME and nvAMD in phase 1 trials at a dose of 1.25mg in 0.05ml. The cost of compounded ziv-aflibercept is much reduced to USD 67 per dose. The 1.25mg dose of ziv-aflibercept is below that recommended for intravitreal injections of aflibercept. However, to the best of our knowledge, there are no data available on the safety of 2mg of ziv-aflibercept, although *in vitro* studies indicate that it is safe. Furthermore, there is no data on the intravitreal administration of ziv-aflibercept in the Ghanaian population to date.

General Aim: To evaluate the safety of 1.25mg and 2mg ziv-aflibercept in Ghanaian population with retinal vascular diseases.

Methodology: This is a prospective, randomised, double masked, phase 1, interventional study. Twenty (20) patients with centre involving DME, ME following RVO, and nvAMD will be assigned to 2 groups: 1.25mg/0.05ml (control) and 2mg/0.08ml ziv- aflibercept and will receive 3 doses of ziv-aflibercept at 4 weekly intervals. Intraocular pressure will be determined 30 minutes following injection and in subsequent visits. Safety data will be collected at days 1and 7 after initiation of treatment, and at 4, 8 and 12 weeks.

Primary outcome measures are ocular safety parameters- including the incidence of pain, blurred vision, raised intraocular pressure, intraocular inflammation and endophthalmitis(eye infection), as well as systemic safety at 4 weeks.

Secondary outcome measures are ocular and systemic safety parameters at 12 weeks, change in BCVA (ETDRS letters), central subfield foveal thickness (CSFT) and central retinal thickness (CRT) as measured on optical coherent tomography (OCT) at 4 and 12 weeks.

Expected Outcome: We seek to evaluate the safety of 2 different doses of ziv-aflibercept in patients with DME, ME following RVO and nvAMD in a Ghanaian population. We expect that there will be no differences in safety between the 2 doses of ziv-aflibercept.

BACKGROUND

INTRODUCTION

Retinal vascular diseases such as diabetic macular edema (DME), neovascular age related macular (nvAMD), and macular edema (ME) following retinal vein occlusions (RVO) are a significant cause of visual impairment in developed countries. They are increasingly becoming important cause of blindness in developing and low-middle income countries including Ghana. (1-3)Vascular endothelial growth factor, which promotes angiogenesis and increases vascular permeability, has been found to play an important role in the pathogenesis of these diseases.(4-6) Ranibizumab (Lucentis; Genentech, San Francisco, California, USA/Novartis, Basel, Switzerland) and aflibercept ((Eylea; Regeneron, Tarrytown, New York, USA) have been approved by the USA FDA and European Medicines Agency (EMA) for the treatment of DME, nvAMD and ME following RVO. Bevacizumab (Avastin, Genentech Inc. USA /Roche, Basel, Switzerland) and ziv-aflibercept (Zaltrap, Sanofi-Aventis US, LLC, Bridgewater, New Jersey, USA and Regeneron Pharmaceuticals, Inc., Tarrytown, New York, USA) have been approved by the USA FDA and EMA for the treatment of colorectal cancers.(7) Bevacizumab (off-label) is the most commonly used anti-VEGF for the treatment of these retinal vascular diseases due to its cost- effectiveness when compounded.

There are concerns about the use of ziv-aflibercept due to potential retinal toxicity from its hyper-osmolality but no safety signals were seen one month after single intravitreal injections into eyes with nvAMD and DME.(8, 9) Ziv-aflibercept which is structurally identical to but cheaper than aflibercept when compounded has been reported to be efficacious and safe for the treatment of DME and nvAMD in phase 1 trials when given in a dose of 1.25mg intravitreally.(8-14) The optimal dose of intravitreal ziv-aflibercept, however, is not known, as

only a single dose of 1.25mg has been studied to date.(8-14) The recommended intravitreal dose of aflibercept, however, is 2mg/0.05ml derived from the VIEW studies. (15) However, based on the earlier studies with aflibercept, the 1.25mg dose of ziv-aflibercept may be inferior to the 2.0mg dose currently given for aflibercept.(16, 17) To date there are no reports on outcomes of 2mg/0.08ml of intravitreal ziv-aflibercept in human eyes with retinal diseases.(10-14) Malik et al has shown that the use of 2mg ziv-aflibercept did not affect the viability of human retinal pigment epithelial cells in vitro although there was a mild reduction in mitochondrial membrane potential.(18) Intravitreal injection of 4mg aflibercept in human eyes with nvAMD was found to significantly improve visual acuity compared to 0.15mg dose and also found to be well tolerated and safe in a phase 1 trial.(19)

As aflibercept is safe when given at 2.0mg intravitreally, we postulate that 2mg/0.08ml ziv-aflibercept is safe and we seek to explore the safety of this dose in patients with retinal diseases in a Ghanaian population.

Rationale/Relevance/Justification.

The magnitude of blindness and visual impairment from retinal vascular diseases is rising in developing and low-middle income countries such as Ghana. This can be attributed to increasing life expectancy and changing life style associated with increasing prevalence of metabolic risk factors such as diabetes mellitus, hypertension, hypercholesterolemia and obesity, and lack of awareness, low priority for, and inadequate eye care services to treat patients with these diseases. Ghana is a low middle-income country.(1-3) The main retinal vascular diseases in Ghana requiring treatment with anti-VEGF are DME, nvAMD, and ME following RVO. The treatment with anti-VEGF is not covered by the National Health Insurance scheme in Ghana. Bevacizumab (used off-label) is the main anti-VEGF used for the treatment of these conditions in Ghana due to its cost effectiveness. Due to individual variability in their response to a particular anti-VEGF the availability of alternative agent and of similar cost to bevacizumab will be useful particularly in patients who are recalcitrant or poor responders or non-responders to bevacizumab. Unfortunately, aflibercept is expensive ($1850 US per dose) and many needy patients in developing countries including Ghana lack the funds to pay for treatment. Just as bevacizumab became a commonly used low-cost alternative to branded anti-VEGF medications, ziv-aflibercept is being investigated as an off-label treatment for various retinal disorders.(8-14) Aflibercept has been shown to be effective in the treatment of retinal vascular diseases at 2.0mg intravitreally, and results in better outcomes than the 0.5mg dose.(15-17) Ziv-aflibercept (Zaltrap) is similar to aflibercept (Eylea), with an identical active molecule in both formulations. The differences

between the formulations are in the concentration of the active ingredient and the excipients.(9, 20) Short term reports have shown that 1.25mg dose of ziv-aflibercept is safe in patients with DME, RVO, and nvAMD, and the cost of treatment is similar to bevacizumab although there is no experience of its use in a Ghanaian population.(8-14, 21, 22) However, based on the earlier studies with aflibercept, the 1.25mg dose of ziv-aflibercept may be inferior to that of 2.0mg dose (similar to the clinically recommended dose of intravitreal aflibercept).(16, 23) Malik et al has shown that the use of 2mg ziv-aflibercept did not affect the viability of human retinal pigment epithelial cells in vitro although there was a mild reduction in mitochondrial membrane potential.(18)

AIM: To evaluate the safety of 1.25mg and 2mg ziv-aflibercept in Ghanaian population with retinal vascular diseases.

Specific Objectives:

1. To determine the safety of intravitreal injections of ziv-aflibercept at 4 and 12 weeks in a Ghanaian population.
2. To measure the visual outcome of treatment with 1.25mg and 2mg ziv-aflibercept in eyes with DME, nvAMD, and ME secondary to RVO at 12 weeks.
3. To measure the anatomic changes using SD-OCT in eyes with DME, nvAMD and ME secondary to RVO at 12 weeks.

LITERATURE REVIEW

The magnitude of blindness and visual impairment from retinal vascular diseases such as DME, RVO and nvAMD is increasing in developing and low middle income countries such as Ghana due to increasing life expectancy and changing life style associated with increasing prevalence of metabolic risk factors such as DM, hypertension, hypercholesterolemia and obesity, and lack of awareness, low priority for, and inadequate eye care services to treat patients with these diseases.(1-3)

Vascular endothelial growth factor (VEGF) plays an important role in the pathogenesis of ME in patients with DME, RVO and nvAMD by increasing the permeability of retinal vessels.(4-6) Anti-VEGF agents such as ranibizumab and bevacizumab bind to VEGF receptors 1 and 2 and prevent all isoforms of VEGF A from binding to these receptors. Aflibercept and ziv-aflibercept are fusion proteins which acts as decoy receptors and binds to all isoforms of VEGF A and VEGF B and placental growth factors 1 and 2. The use of these anti-VEGF have been shown to improve visual acuity or prevent severe visual loss in patients with ME from DM, RVO and nvAMD.(5, 24-28) The CATT and IVAN trials have reported that ranibizumab and bevacizumab have similar efficacy in patients with nvAMD. (26, 27) The VIEW 1 and 2 studies have shown that aflibercept has similar efficacy as ranibizumab in nvAMD. (15, 23) The Diabetic Retinopathy Clinical Research Network (DRCR.net) have shown that ranibizumab and aflibercept had similar efficacy which was superior to bevacizumab in patients with DME at 1 year, although the mean change in visual acuity letter score was better with aflibercept among patients with visual acuity of 20/50 or worse.(28) This difference between aflibercept and ranibizumab was, however, not sustained at 2 years.(29) The CRUISE, COPERNICUS, and GALILEO trials, and Epstein et al have shown that ranibizumab, aflibercept, and bevacizumab, were safe and effective in the treatment of ME from RVO.(24, 25, 30, 31) Rajagopal et al reported that bevacizumab and ranibizumab had similar efficacy at 6 months in patients with RVO in the CRAVE study. (32)

Ziv-aflibercept has been reported to be safe in vitro and in vivo, and in phase 1 trials of patients with retinal vascular diseases. (7-13) Preliminary results have reported that 1.25 mg ziv-aflibercept was safe and effective in nvAMD and DME at 3 months.(12-14) There are no reports on the efficacy and safety of 1.25mg ziv-aflibercept beyond 3 months in patients with nvAMD. In a pilot study of 7 naïve eyes with DME treated with six 4-weekly injections of 1.25mg ziv-aflibercept, the mean BCVA improved from 20/120 at baseline to 20/33 at 24 weeks ($p<0.001$), with significant reduction in mean macular thickness on optical coherence tomography(OCT).(10) There were no adverse events reported.(10) Similarly, Chhablani et

al and Paulose et al have provided pilot data on the safety and efficacy of ziv-aflibercept in patients with ME secondary to RVO.(21, 22)Ranibizumab and aflibercept have been approved by the USA FDA for the treatment of ME in patients with retinal diseases such as DME, RVO and nvAMD. However, the cost of treatment per dose of intravitreal ranibizumab (USD1950) and aflibercept (USD 1961) are quite expensive and beyond the reach of majority of patients with retinal diseases in developing countries. The cost per intravitreal injection of bevacizumab (USD 67) is much lower and widely used off-label for the treatment of retinal diseases worldwide. The cost of treatment per dose of intravitreal ziv-aflibercept is similar to that of bevacizumab and is being investigated as an off-label treatment for various retinal disorders.(8-14, 21, 22) The recommended dose of intravitreal aflibercept is 2mg/0.05ml. To date, the dose of intravitreal ziv-aflibercept reported in previous studies in human eyes is 1.25mg/0.05ml and has been found to be safe (7, 8). A single case report of 1.75mg/0.07ml ziv-aflibercept has been found to improve the electroretinogram (ERG) and microperimetry of an eye with resistant choroidal neovascular membrane (CNVM) due to AMD. (33) The use of 2mg ziv-aflibercept has not been reported in literature. There are concerns about the use of ziv-aflibercept due to hyper-osmolality, and potentially higher elevation of intraocular pressure (IOP), as a higher volume of 0.08ml is required to achieve that dose. Intravitreal injections of pegaptanib (Macugen, Pfizer), the first anti-VEGF agent licensed for intraocular injection was approved to be delivered at a dose of 0.3mg in 0.09ml of volume.(34) Although there was transient elevation of intraocular pressure, this did not translate into serious adverse events. (34) Pegaptanib use has now been superseded by more efficacious anti-VEGF agents. The potential retinal toxicity from hyper-osmolality of ziv-aflibercept has been refuted by several short term studies.(8-14, 21, 22, 33) The calculated osmolality of 2mg of ziv-aflibercept in 4ml of human vitreous is 320mosm/Kg which is within physiological range and does not affect the retina.(9, 35, 36) Malik et al has shown that the use of 2mg ziv-aflibercept did not affect the viability of human retinal pigment epithelial cells in vitro although there was a mild reduction in mitochondrial membrane potential. (18) Similarly, de Oliveira Dias (2015) has reported that ziv-aflibercept was safe in the rabbit eye when given intravitreally in doses up to 25mg/ml.(37)

Based on the earlier studies with aflibercept where a differential dose effect was shown, the 1.25mg dose of ziv-aflibercept may be inferior to a 2.0mg dose (similar to that of the 2.0mg dose currently given for aflibercept). As such, we seek to evaluate the safety of this higher dose (2.0mg) of intravitreal ziv-aflibercept in a Ghanaian population.

METHODOLOGY

Study Design: Prospective randomised double mask Phase I interventional study.

Study setting: The infrastructure of the outpatient department (OPD) of the Eye Centre, Korle Bu Teaching Hospital include(number) –Consulting rooms (10), minor procedures/treatment rooms(2), Diagnostic rooms(3), refraction room(2), Low vision room(1), Laser treatment room(1) and optical work shop(1). The centre also has admission wards, private wing comprising of 3 consulting room, Research office, Library, Conference room and administrative offices. Each consulting room is equipped with the following Slit lamp Biomicroscope (Haag Strait model) and Goldman applanation tonometer. There are also indirect ophthalmoscopes (5) and early treatment diabetic retinopathy study (ETDRS) acuity charts (literate chart-3 and illiterate chart-1) used for research purposes, examination couch (2), gonioscopy lenses, and Volk 66D, 90D and 78D lenses. The diagnostic/investigation rooms are equipped with the following: Spectral Domain OCT (3D OCT-2000, Topcon, Tokyo, Japan), Fundus Camera (Carl Zeiss Inc. Jena, Germany) with capability of taken fundus photograph and performing fluorescein angiography, automated visual field analyser, Ultrasound Scanning machine(2), and keratometer(2). The minor procedure room is equipped with operating microscope, operating table, autoclave machine and a side room containing refrigerator for storage of intravitreal drugs at temperature of 4 degrees Celsius. The research office has desk and secured cabinets for safe keeping of records of patients and one consulting room in the private patients' wing has been earmarked for research purposes only. There is a day care room equipped with sphygmomanometers (Blanket MK-3, Accoson, England), glucometer machine (Biosensor Inc., Gyeonggi-do, Korea), and thermometers. Wednesdays are designated clinic days for retina disorders although patients with retina disorders are seen from Mondays to Fridays. Personnel of retina unit: retina specialist (3), residents/medical officer (3), image technician (1) and an ophthalmic nurse (assisted by other ophthalmic nurses).

Study Duration: 5 months. From **1st November 2017 to 31st March, 2018** and 2 months for trial close out procedures.

Target Population: all patients with DME, macular edema from RVO, and nvAMD attending the Korle Bu Teaching Hospital Retina Clinic.

Participants:

Patients will be recruited based on clinical suspicion and will be assessed for eligibility as outlined below:

Inclusion criteria:

Age 18 years or older

Meets diagnostic criteria for DM, RVO and active nvAMD (Appendix I)

Treatment naïve

Understands and willing to sign consent form

Able to comply with clinic visits

Centre involving ME in patients with diabetes mellitus and RVO with retinal thickness >300um using SD- OCT

Best corrected visual acuity (BCVA) of 6/12 or worse

Exclusion criteria:

Glaucoma or raised intraocular pressure (>21mmHg)

Intraocular surgery within 3 months in the study eye

History of uveitis

Pregnant or breastfeeding mother

Renal failure on dialysis or had kidney transplant

Allergy to active drug or excipients

Cardiovascular events such as myocardial infarction or CVA

Eye infections such blepharitis, dacryocystitis, conjunctivitis or keratitis.

Unwilling to sign consent form or to come for follow up visits

Myopia ≥-6.0 Dioptres

Drugs Used:

Ziv-aflibercept and aflibercept are fusion proteins comprising of the Fc portion of human immunoglobin IgG1 and extracellular matrix domains of VEGF receptors 1 and 2. They act as decoy receptors by binding to circulating VEGF- A and B and placental growth factor. Ziv-aflibercept is an 115kDa molecule manufactured from Chinese hamster ovary cells.(9) The molecular structure of ziv-aflibercept is identical to aflibercept but are different because they undergo different purification processes and whilst aflibercept is iso-osmotic (300mosm/kg) ziv-aflibercept is hyperosmotic (1000mosm/kg) due to addition of higher concentration of sucrose.(9, 20) Ziv-aflibercept is packaged as 25mg/ml of ziv-aflibercept in polysorbate 20 (0.1%), sodium citrate (5 mM), sodium phosphate (5 mM) and sucrose (20%), in water for Injection USP, at a pH of 6.2 and aflibercept as 40mg/ml aflibercept in 10 mM sodium phosphate, 40 mM sodium chloride, 0.03% polysorbate 20, and 5% sucrose, pH 6.2.(9) Aflibercept has been approved by the USA FDA for ocular use whilst ziv-aflibercept was

approved for the treatment of metastatic colorectal cancers and other cancers.(7) The standard dose of intravitreal aflibercept is 2mg/0.05ml.(15) The usual (standard) dose of intravitreal ziv-aflibercept reported in short term series is 1.25mg/0.05ml.(8-13, 22) 1.75mg/0.07ml ziv-aflibercept has been found to improve the electroretinogram (ERG) and microperimetry of an eye with resistant CNVM due to AMD in one case report.(33)

Ziv-aflibercept is supplied in single use vials of 100mg in 4ml or 200mg in 8ml. The 100mg in 4ml vial of ziv-aflibercept will be used in this study. The vials are stored at 4 degrees centigrade and 1.25mg in 0.05ml and 2mg in 0.08ml of ziv-aflibercept will be drawn using 25-gauge needle, micro filter and 1ml syringe with luer lock tip. The drawn ziv-aflibercept in 1ml syringes will be used within 2 weeks of initial preparation.(9)

Randomization process

Procedures to be used (Sampling Methods)

At enrolment, a detailed medical history will be obtained from all eligible patients including age, sex, duration of symptoms, history of hypertension, diabetes mellitus, hyperlipidaemia, cigarette smoking, history of current medications, diagnosis and previous treatments, and comprehensive ocular examinations including: best corrected visual acuity (BCVA) measurement using a standardised early treatment diabetic retinopathy study(ETDRS) visual acuity chart and recorded as Visual acuity using ETDRS chart and recorded as logarithm of minimum angle of resolution(logMAR), intraocular pressure(IOP) measurement using Goldman's applanation tonometer, slit lamp biomicroscopy (Haag Strait model) examination of the anterior segment and posterior segment examination with the aid of Volk 90D or 78D lenses. If both eyes are eligible, only the eye with the worse visual acuity will be recruited into the study**.** Comprehensive ocular examination will be repeated at day 1 and 7 after the initial anti-VEGF injection and in all subsequent visits. All patients will undergo fundus photography (ZEISS 450 FUNDUS CAMERA, ZEISS INC. JENA, GERMANY), Fluorescein angiography (ZEISS 450 FUNDUS CAMERA, ZEISS INC. JENA, GERMANY)) and spectral domain optical coherence tomography (SD-OCT) (TOPCON 2000, TOKYO, JAPAN) at baseline. Fundus photography and SD-OCT will be repeated on days 7 and 28 and in all subsequent visits as per study protocol.

Systemic arterial blood pressure, fasting lipids and fasting blood sugar will be measured at baseline. Systemic arterial blood pressure and fasting blood sugar at visits on days 1, 7 and 28. After obtaining informed consent, all eligible patients will be randomly assigned to either 1.25mg or 2mg ziv-aflibercept using simple random sampling which involves picking 2

labelled cards from an envelope by an individual independent of the study investigators, but the treatment is concealed to the patients.

Preparations of Ziv-aflibercept injections

To minimize the risk of repeated puncture of the vial, the vial containing 100mg/4ml of ziv-aflibercept will be punctured once under the laminar air flow system at the pharmacy manufacturing unit of KBTH and withdrawn using 5μ microfilter in 0.1ml and 0.15ml aliquots into 1 ml syringes, labelled and each syringe kept in separate sterile plastic wrappers (***Eye Drape plus***, ***Aurolab, India***) and are immediately stored at 4 degrees Celsius. Two (2) syringes containing the withdrawn samples shall be cultured on chocolate agar and sabouraud agar by MDS-LANCET laboratories ltd. Negative culture report shall be received before the remaining samples will be released for injection. The first syringe and the last drawn product shall be sent for microbiologic studies. Each time the vial is to be used we shall inspect the syringe physically for contamination or change in colour. The stored ziv-aflibercept in 1ml syringes shall be used within 2 weeks from the date of preparation. Omek et al (2008) found that storage and multiple use of anti-VEGF from single-use vials does not seem to result in microbial contamination for up to 15 days from initial use.

Intravitreal injection procedures

Standard precautions relating to intravitreal injections shall be observed. Patients will be pooled together for Injections once per week. An unmasked certified physician, will give the intravitreal injections. The intravitreal injection will be done using a sterile technique. Topical anaesthetic agent proparacaine and 5% povidone iodine will be instilled into the conjunctival cul-de sac and periocular skin, eyelids and lashes will be cleaned using 10% povidone iodine. The eye will be draped and the injection given into the mid vitreous cavity 4 mm or 3.5mm posterior to the limbus in phakic and pseudophakic eyes, respectively. Hand motion vision is checked and confirmed to be present. No topical antibiotics will be given prior to, during or after each injection. Intraocular pressure (IOP) will be checked at 30 minutes after intravitreal injection and at subsequent visits (on day 1, 7 and 28) by the examining physician (IZB) who will be masked to the treatment doses. The examining physician (IZB) will also assess each patient for ocular and systemic adverse events on days 1, 7 and 28 after initial injection. Ocular adverse events include incidence of raised intraocular pressure (>21mmHg), corneal abrasions, cataracts, and intraocular inflammation. Systemic evaluations include systemic arterial blood pressure, fasting lipids and fasting blood sugar measured at baseline and at visits on day 1 and 7. Systemic arterial blood pressure will be measured in all subsequent visits. Each patient will receive 3 doses of ziv-aflibercept at 4 weekly intervals. At 12 weeks, the anatomic response of the patients to anti-VEGF will be assessed by an independent investigator (WMA) based on

the change macular morphology using SD-OCT. Any disparity in grading, as necessary, will be adjudicated by a panel of 3 investigators (WMA/IZB/KAA).

SCHEDULE OF EVENTS TABLE

	Baseline	Day 0	Day 0 30 min	Day 1	Day 7	Day 28	1 month	2 months	3 months
Informed consent	×								
demographics	×								
Review medical history	×								
Ocular examination	×			×	×	×	×	×	×
Intraocular pressure check	×		×	×	×	×	×	×	×
Fluorescein angiography	×								
Fundus photo	×				x	×	×	×	×
SD-OCT	×				x	×	×	×	×
Blood pressure	×			×	×	×	×	×	×
Fasting blood sugar	×			×	×				
Fasting lipids	×			×	×				
Adverse event evaluation				×	×	×	×	×	x
Randomization	×								
Administer IVZ injection		x					x	x	

Sample size: 20 eyes will be included in this study. Only One eye per patient will be included in the study. Where both eyes are eligible, the eye with the worse visual acuity will be selected for the study.

Outcome measures

To date, no serious ocular or systemic adverse event have been reported with intravitreal ziv-aflibercept injection (8-14, 21, 22, 33) although these studies are short-term with small numbers. The only minor adverse event related to intravitreal injection of ziv-aflibercept was conjunctival thinning which occurred in 1 eye of 12 patients with nvAMD and resolved on administration of topical antibiotics.(8) In a comprehensive review(Meta-analysis) of the ocular and systemic safety of intravitreal aflibercept in 10 phase II and phase III clinical trials of patients with DME, ME following retinal vein occlusion and nvAMD using person-years at risk methodology, Kitchens et al demonstrated that eyes treated with intravitreal aflibercept injection were not at increased risk of intraocular inflammation or endophthalmitis, had lower rates of hypertension and differences observed with serious adverse events(SAEs) and APTC-ATEs were consistent with chance variations.(38) They concluded that the rate of ocular and systemic adverse events with intravitreal aflibercept injection (IAI) were similar to controls and across disease states. (38) In an analysis of 2 phase III trials using 2mg dose of aflibercept every 4weeks, Freund et al reported that the proportion of eyes with IOP>21mmHG at 96 weeks visit was 14.2%.(39)

The primary outcome measure to be evaluated in this study is safety of intravitreal ziv-aflibercept at 4 weeks. Ocular toxicity will be assessed based on the number of ocular adverse events such as blurred vision (mild-loss of 0.1logMAR, moderate- loss of 0.2 logMAR and severe- ≥ 0.3 logMAR), raised intraocular pressure (>21mmHg), corneal abrasions, cataract, intraocular inflammation and *endophthalmitis (eye infections)* using a predesigned questionnaire (Appendix III case report form) and SD-OCT findings.

Corneal abrasion- An area of corneal epithelial defect assessed on slit lamp biomicroscopy after instillation of fluorescein eye drops, before any applanation tonometry is performed.

Cataract- presence of Nuclear sclerosis, cortical, posterior subcapsular, anterior capsular. This will be assessed using slit lamp biomicroscope after pupil dilatation.

Intraocular inflammation/endophthalmitis will be assessed by the degree of anterior camber cell, flare, aqueous fibrin and hypopyon, vitreous cells and haze(vitritis), vasculitis and choroiditis using the standardised uveitis nomenclature (SUN) working group classification.(40) Inflammation in the anterior segment is determined by assessing the cell count and flare at baseline and at subsequent visits. The assessment is based on number of cells in the anterior chamber(AC) in a 2mm long X 1mm wide slit beam with maximal light intensity and magnification. The slit beam is directed at 45 degrees to the iris plane. Grading of cell and flare will be done according to the grading scale in Appendix III. Vitritis will be

assessed using the grading scale in appendix III- table2. The definitive proof of endophthalmitis is dependent on vitreous biopsy and microbiological evaluation (microscopy, culture).

Posterior vitreous detachment(PVD)- is a condition where the gel-like substance that occupies the space between the retina and the lens of the eye liquefies and separates from the retina. PVD is diagnosed by slit-lamp biomicroscopy, which will usually show a prominent plane defining the posterior vitreous face. The plane is seen in the form of inverse S-shaped in the anterior vitreous cavity. The presence of a glial annulus in the vitreous cavity (Weiss ring) is strong evidence of PVD. The presence of PVD will be assessed by slit lamp biomicroscopy, fundus biomicroscopy with 90D/78D lenses and indirect ophthalmoscopy with +20D lens. In the Presence of PVD, peripheral retinal examination with indirect ophthalmoscope with scleral indentation is mandatory.

Pain severity scale- Eye pain will be assessed using the eye sensation scale (Caudle et al. 2007) The eye sensation scale is divided into 5 categories : none, mild, moderate, severe and extreme(Appendix III – CRF) .

Systemic adverse events will be assessed based on systemic evaluation using predesigned questionnaire (Appendix III). Systemic adverse events will be initially assessed based on history and confirmation from patient’s physician or from the physician for this study (EK).

Secondary outcome measures are occurrence of ocular and systemic adverse events at 12 weeks (Appendix I and II) such as IOP>25mmHg, increased IOP > 10mmHg and >15mmHg from baseline, change in BCVA (logMAR), BCVA improvement of at least 0.1 logMAR, BCVA improvement of at least 0.2 logMAR, BCVA improvement of at least 0.3 logMAR, BCVA worsening of at least 0.3 logMAR, central subfield foveal thickness (CSFT) and central retinal thickness (CRT) using SD-OCT at 4 and 12 weeks.

The toxicity grading Scale that will be used to assess the safety of ziv-aflibercept will be based on the severity of the outcome measures outlined above and include mild, moderate, severe and potentially life-threatening and death (Appendix IV- Toxicity grading scale) scale

Data Handling

Patients’ data will be recorded using a pre-designed questionnaire. The records of participating patients will be kept in a secured cabinet with only the principal investigator having access to the cabinet. The Senior research assistant will be responsible for data entry. Computer

designated for data entry will be password protected and only the data entry manager and principal investigator will have access to the computer. The use of the computer for internet surfing will be prohibited. All data entry will be proofed for accuracy by the principal investigator by visual proofing of the case report form(CRF) and cross-referencing with source documents (thus, SD-OCT, Fundus photographs, fasting blood sugar and lipid print outs). All data will be captured and cleaned using predesigned Microsoft excel version 2015 by the Senior research assistant and principal investigator. Patients' data will be handled by strict adherence to the data protection act and the Health insurance portability and accountability act. Scanned and keyed data will be saved in a designated computer and backed up onto external hard disc drive and flash disc every 2 weeks. The only data that will be transmitted via a secured internet website are Fundus photographs, fundus fluorescein angiograms and SD-OCT for analysis by independent investigator (WMA). The images will be anonymised by given each image unique codes before transmission via the University of Nottingham Website to the independent investigator. The independent investigator shall establish and maintain the confidentiality and security measures necessary to ensure the integrity of the transmitted data in accordance with measures specified in the data protection Act. At the close of the trial the principal investigator shall retain control of both manual and electronic records and ensure proper archiving of the records. All relevant electronic records shall be stored in an external hard disc drive and the case report forms, consent forms, study files, fundus photographs and SD-OCT reports shall be archived at the medical records department in a secured cabinet for 2 years after the close of the trial. All other data related to the trial stored in the designated computer shall be destroyed.

Statistical Analysis

All statistical analyses for this study will be done using STATA 13 (Statacorp, Texas, USA). The frequencies of ocular and systemic adverse events and serious adverse events will be computed. Continuous variables will be presented as mean and standard deviation. Pre- and post-injection changes in BCVA, intra ocular pressure, and central subfield foveal thickness will be compared using paired t-test. ANOVA and MANOVA will be used to assess the repeated measures at times 0, 4, 8 and 12 weeks.A P value<0.05 will be considered statistically significant.

STATISTICAL PLAN

The unmasked statistician(EK) will analyse the data based on the following schedules:

1. after 50% of subjects have been recruited and followed for 1 month
2. after 100% of subjects have been recruited and followed up for 1 month.

3. After 100% of subjects have been recruited and followed up for 3 months

The interim analyses will include demographic and baseline characteristics by group, analysis of subjects screened, enrolled, active, completed and terminated, and primary outcome endpoints including the incidence of pain, blurred vision, raised intraocular pressure, cataract, intraocular inflammation, endophthalmitis, and systemic adverse events by group. The data will be presented by masked treatment groups to the data and safety monitoring board (DSMB) and a decision on early stopping or continuation of the trial taken in the event of occurrence of large differences or serious adverse events observed in the interim analysis.

DISSEMINATION OF RESULTS

The outcome of this study will be presented in national and international conferences and published in peer reviewed journals.

ETHICAL ISSUES

This research will be conducted in accordance with the Declaration of Helsinki on human subjects. The proposal will be submitted to the Institutional Review Board (IRB) of the Korle Bu Teaching Hospital (KBTH) for approval prior to commencement of the study. License/clearance for use of this drug will be obtained from FDA Ghana. Product liability insurance has been secured for all participants. The records of patients will be assigned codes. Participants' information will be treated as confidential. To ensure confidentiality, names of participants will not appear on any write-up. Patients will only be eligible to participate after obtaining informed consent (Appendix II).

The safety issues to be considered in the study to ensure protection of patients include: adverse events, serious adverse events and unanticipated problems. Adverse event means any untoward medical occurrence associated with the use of an intervention in humans, whether or not considered intervention-related. A suspected adverse reaction is considered "serious" if, in the view of the investigator it results in any of the following outcomes: death, a life-threatening adverse event, inpatient hospitalization or prolongation of existing hospitalization, a persistent or significant incapacity or substantial disruption of the ability to conduct normal life functions.

An unanticipated problem involving risks to patients include any incident that is Unexpected in terms of nature, severity, or frequency given the research procedures that are described in the protocol-related documents.

The anticipated adverse events (AE) and serious adverse events (SAEs) related to intravitreal aflibercept injection are shown in Appendix II (ocular and systemic adverse events) and include blurred vison, raised intraocular pressure, sterile intraocular inflammation and endophthalmitis. The principal investigator and Study physician (EK) will be responsible for determining whether an AE is expected or unexpected. An AE will be considered unexpected if the nature, severity, or frequency of the event is not consistent with the risk information previously described. All AEs including local and systemic reactions not meeting the criteria for SAEs will be captured on the appropriate AE report form. Information to be collected includes event description, time of onset, clinician's assessment of severity, relationship to study product (assessed by PI and co-investigators), and time of resolution/stabilization of the event (Appendix III-CRF). All AEs occurring while on study will be documented appropriately regardless of relationship. All AEs will be followed to adequate resolution. Unanticipated problems will be recorded in the data collection system throughout the study. Changes in the severity of an AE will be documented to allow an assessment of the duration of the event at each level of severity to be performed. The PI will record all reportable events with start dates occurring any time after informed consent is obtained until 7 (for non-serious AEs) or 30 days (for SAEs) after the last day of study participation. At each study visit, the PI and co-investigators will inquire about the occurrence of AE/SAEs since the last visit. Events will be followed for outcome information until resolution or stabilization.

ADVERSE EVENT REPORTING-

On suspicion of adverse events, the event will be recorded in the adverse event report book and the principal investigator will be notified immediately. Investigations will be carried out to determine the duration, likely cause of the adverse event, mitigating measures and a report generated by the principal investigator and unmask statistician. The members of the Data and Safety Monitoring Board (DSMB) will also be informed immediately and a meeting convened to determine the seriousness of the adverse event, the likely cause and a decision taken on modifications of the trial. A final report on the specific adverse event will be submitted to the chairman of the KBTH-IRB and the director of FDA Ghana will be duly informed on the occurrence of this adverse event and the decision taken by the DSMB regarding this issue.

When adverse event is considered serious, a SAEs report form will be completed by the principal investigator and unmasked statistician and the DSMB will be notified within 24hrs.

The study clinician will complete a SAE Form within the following timelines: • All deaths and immediately life-threatening events, whether related or unrelated, will be recorded on the SAE Form and submitted to DSMB within 24hrs awareness. Other SAEs regardless of relationship, will be submitted to the DSMB within 48hrs of awareness. All SAEs will be followed until satisfactory resolution. Other supporting documentation of the event may be requested by the DSMB and should be provided within 48 hours. The PI will be responsible for notifying FDA of any unexpected fatal or life-threatening suspected adverse reaction within 48hrs after the PI's initial receipt of the information. In the event of occurrence of eye pain, the affected eye will be examined to exclude raised intraocular pressure and intraocular inflammation and the pain will be relieved with topical analgesic agents. Patient will be followed up for 7 days after cessation of the adverse event. In the presence of sterile inflammation topical corticosteroids will be prescribed and patient monitored daily for response to treatment or progression of the inflammation. When endophthalmitis is suspected, vitreous specimen will be taken for microbiology and intravitreal injection of antibiotics will be given followed by topical antibiotics and corticosteroids, patient monitored daily and treatment revised based on patients' response to treatment.

The rescue treatment that will be given to participants in the event of exacerbation of the condition whilst on the IP and / or when there are symptoms of toxicity after IP administration will be bevacizumab. Although the use of bevacizumab is also off-label, its safety and efficacy has been established in land mark clinical trials. (24-31)

Termination rules:

The study will be halted according to the following criteria:

1.Three adverse events classified as severe (grade 3) have been reported

2.One potentially life -threatening adverse event (grade 4) have been reported

3. Two or more cases of endophthalmitis have been reported from the same vial of injection.

4.Large differences in the mean change in acuity (≥ 0.3logMAR) between the groups

5.Serious violation/deviation deem to significantly affect the overall study outcome as determined by DSMB (protocol violation log Appendix VIII).

The stopping rules for a particular participant include the following: participant has completed the study, occurrence of serious adverse event, lost to follow-up, non-compliant participant (especially if 4week visit missed), withdrawal of consent, and death. In the event of stopping of participant from the trial, an off study form(Appendix III CRF) will be completed and adverse event reporting form (Appendix III) will be completed if this due to occurrence of AE/SAE. The primary outcome measure will be determined at 4 weeks. If a patient leaves the trial after 4week visit the patients' data will be included in the analysis. If a patient leaves the trial prior to 4 weeks after enrolment that particular patient shall be replaced by another patient and their data shall not be included in the final analysis. The original randomisation process shall be utilised in the replacement of the patient if recruitment is not completed. No replacement will be allowed if enrolment of the required number of patients is completed. Where a patient stops the trial, the patient shall be followed for 30 days after the last injection or after resolution of adverse events. The data that shall be collected for every patient stopping the trial include Date of last injection, date off study, reason off study and/or adverse events (Appendix III)

The principal investigator will inform the DSMB members within 24hrs of occurrence of serious adverse event and will provide the DSMB with AE listing reports. The DSMB will convene an ad hoc meeting by teleconference or in writing within 48hrs. The DSMB will provide recommendations for proceeding with the study to the principal investigator. The principal investigator will inform the KBTH-IRB and FDA of the temporary halt and the disposition of the study within 48 hours of receiving the report from the DSMB.

PROTOCOL VIOLATIONS/DEVIATION

In the event of occurrence of protocol violation, a protocol violation log (appendix VIII) will be completed and the DSMB informed immediately. The DSMB shall convene a meeting within 48hrs to determine the effect of the protocol violation on the conduct and outcome of the study. The decision of the DSMB shall be communicated to the KBTH-IRB and FDA within 48hrs.

Data and Safety Monitoring Board (DSMB)

A data and safety monitoring board (DSMB) comprising of three members has been constituted. The members include Dr. Edith Dogbe (Chairperson), JCB Dr. Dakubo (member) and Dr. NN Tagoe (member). Dr. Richard Awa (ophthalmology resident) will serve as the executive secretary to the DSMB. The executive secretary shall have no voting right. He will attend only the open session and Confidentiality shall be strictly observed.

The DMSB will operate under the rules of an approved charter that will be written and reviewed at the organizational meeting of the DSMB. The proposed DSMB charter is as shown in Appendix IV. The DSMB shall meet before patient enrolment, 1 month after 50% of patient enrolment and at 3 months after 100% of patient enrolment or at any time as deemed necessary. Monitoring for this study will be performed by the DSMB to ensure that the rights and well-being of the enrolled patients are protected, that the reported trial data are accurate, complete, and verifiable, and that the conduct of the trial is in compliance with the currently approved protocol, with Good Clinical Practice, and with applicable FDA Ghana requirements.

TIMELINES/WORK SCHEDULE

This study will be carried out over a period of 24 weeks (***November 2017-March 2018***)

Pre-study Period (November 2016 to **October** 2017)

- development of research proposal and literature review- PI and Co-investigators
- Obtain clearance from ethics committee- PI
- Obtain clearance from FDA Ghana- PI.
- development of questionnaire- PI

November 2017- ***January 2018***- Patient recruitment, Intravitreal injections, Data collection and processing- PI and co-investigators.

February and March 2018- study close out procedures, data analysis and manuscript write – up and Presentation of results/publication.

See below a Gantt chart illustrating different activities versus time frames for expected completion.

Activity **Person** **Duration/Time**

(November 2016- march 2018)

Nov. 16-Oct.17 Nov.17- Jan. 18 Feb18-Mar.18

Proposal write up PI and Co investigators

Ethical Review Board KBTH-IRB, FDA

Patient recruitment, PI and Co investigators

Data collection and processing PI

Data analysis PI and Co investigators

Results compilation PI and Co investigators

Data close out procedures

And manuscript write up PI and Co investigators

<u>PERSONNEL OF THE STUDY TEAM</u>

**Role in Study**	**Name of Personnel**	**Functions**	**Percentage Effort**
**Principal Investigator**	I.Z Braimah	Proposal writing, patient recruitment, intravitreal injection, follow-up review, write up, monitoring & evaluation.	70%
**Co-investigator**	S. K. Akafo	Review of proposal, patient recruitment, intravitreal injection, follow-up review, write up/publication	20%

**Co-investigator**	K.N, Amissah-Arthur	Review of proposal, patient recruitment, intravitreal injection, follow-up review, write up/publication	20%
**Co-investigator**	E. Kenu	monitoring of systemic safety, Statistical analysis and interpretation of data, write up/publication	40%
**Co-investigator**	W. M. Amoaku	Review of proposal, image analysis, independent monitoring of outcome measures, write up/publication,	50%
**Statistician**	A.E Yawson	Statistical analysis and interpretation of data, write up/publication	30%
**Image Technician**	*E.. Ahiabor*	fundus photography, FA and SD-OCT	20%
**Optometrist**	S. Asiedu	refraction	10%
**Senior** Research assistant	B. Abaidoo	Patient recruitment, Data entry	20%

BUDGET AND LOGISTICS

Item	***Cost (GH¢)***
A4sheets(2 rims)	40.00
FLASH DISC	40.00
EXTERNAL HARD DRIVE 500GB	350.00
Printing of proposal	50.00
Processing Fee (KBTH-IRB)	200
Printing of Questionnaire/consent form	100

Intravitreal injection- Zaltrap 3 vials	4800.00
Fluorescein(Aurolab)- 20 vials	400
Fasting blood sugar and lipids	1000
Treatment of Adverse Events	1500
Transport for participants	500.0
Printing of write-up	20.00
Contingency (5% total sum)	370.00
**GRAND TOTAL**	**9450.00**

JUSTIFICATION OF BUDGET LINES:

1. **Equipment.** The ETDRS Charts are the standard visual acuity test charts used for assessment of visual acuity in eye research. The Eye Centre has 4 illuminated ETDRS charts (3 literate and 1 illiterate) is essential for assessment of the secondary outcome measure (Best corrected visual acuity) of this study. The department has available Spectral Domain Optical Coherence Tomography (TOPCON 2000, Tokyo, Japan) and Zeiss 450 fundus Camera (Zeiss, Jena, Germany). These machines will be used for taken cross sectional images of the macular (2 D and 3 d scans), fundus photography, and fluorescein angiography essential for assessment of function and morphology of the macular in patients with retinal vascular diseases. These investigations are essential for establishing diagnosis, monitoring progression of disease and their response to treatment with the anti-VEGF drugs. The investigations will be done free of cost except for provision of consumables such as fluorescein dye for fluorescein angiography.
2. **Consumables.** Total Budget GHC 6200. A maximum of 40 injections can be obtained from each vial of ziv-aflibercept. We expect to give 60 injections over the study period. A vial of ziv-aflibercept once opened can only be used for 4 weeks and the remaining drug discarded. We will be using about 3 vials of ziv-aflibercept over the study period. Each patient will be receiving 3 ziv-aflibercept injection over the study period. Total cost for the 3 vials of ziv-aflibercept is GHC 4800. The cost of 20 vials of fluorescein to be used for fluorescein angiography is GHC 400. The projected cost for Fasting blood sugar and Fasting lipids is GHC 1000.
3. **Office/Administrative Expenditure:** Total budget GHC 2800. To ensure all activities planned are executed, all records properly documented, secured and protected the following stationary will be purchased- A4 sheets, printer toner, Flash disc, 500Giga Byte external hard

drive. GHC 1500 has been budgeted for treatment of adverse events and GHC 500 for transportation of participants.

4. **Contingency-** 5% of the total sum required for the project will be required to meet certain contingencies such as inflation and upward price adjustment of inputs.

References:

1.Dalal S, Beunza JJ, Volmink J, Adebamowo C, Bajunirwe F, Njelekela M, et al. Non-communicable diseases in sub-Saharan Africa: what we know now. *International journal of epidemiology*. 2011;40(4):885-901.

2.Nazimul H RK, Anjli H. Trend of Retinal diseases in Developing Countries. *Expert Rev Ophthalmol 2008*;23(1):43-50.

3.Pascolini D, Mariotti SP. Global estimates of visual impairment: 2010. *Br J Ophthalmol.* 2012;96(5):614-8.

4.Hera R, Keramidas M, Peoc'h M, Mouillon M, Romanet JP, Feige JJ. Expression of VEGF and angiopoietins in subfoveal membranes from patients with age-related macular degeneration. *Am J Ophthalmol.* 2005;139(4):589-96.

5.Ho AC, Scott IU, Kim SJ, Brown GC, Brown MM, Ip MS, et al. Anti-vascular endothelial growth factor pharmacotherapy for diabetic macular edema: a report by the American Academy of Ophthalmology. *Ophthalmology.* 2012;119(10):2179-88.

6.Wang S, Park JK, Duh EJ. Novel targets against retinal angiogenesis in diabetic retinopathy. *Current diabetes reports.* 2012;12(4):355-63.

7.Chung C, Pherwani N. Ziv-aflibercept: a novel angiogenesis inhibitor for the treatment of metastatic colorectal cancer. *American journal of health-system pharmacy : AJHP* : official journal of the American Society of Health-System Pharmacists. 2013;70(21):1887-96.

8.Chhablani J, Narayanan R, Mathai A, Yogi R, Stewart M. SHORT-TERM SAFETY PROFILE OF INTRAVITREAL ZIV-AFLIBERCEPT. *Retina.* 2016;36(6):1126-31.

9.Mansour AM, Al-Ghadban SI, Yunis MH, El-Sabban ME. Ziv-aflibercept in macular disease. *Br J Ophthalmol.* 2015;99(8):1055-9.

10.Andrade GC, Dias JR, Maia A, Farah ME, Meyer CH, Rodrigues EB. INTRAVITREAL INJECTIONS OF ZIV-AFLIBERCEPT FOR DIABETIC MACULAR EDEMA: A Pilot Study. *Retina*. 2016.

11.Ashraf M, Souka AA, El Kayal H, El Manhaly M, Abdallah MH. Three-month outcomes of ziv-aflibercept in the treatment of diabetic macular oedema. *Acta ophthalmologica.* 2016.

12.Mansour AM, Chhablani J, Antonios RS, Yogi R, Younis MH, Dakroub R, et al. Three-month outcome of ziv-aflibercept for exudative age-related macular degeneration. *Br J Ophthalmol.* 2016.

13.Mansour AM, Dedhia C, Chhablani J. Three-month outcome of intravitreal ziv-aflibercept in eyes with diabetic macular oedema. *Br J Ophthalmol.* 2016.

14.Marashi A. Three-Month Outcome of Ziv-Aflibercept for Diabetic Macular Edema. *Advances in ophthalmology & visual system*. 2016;4(3).

15.Schmidt-Erfurth U, Kaiser PK, Korobelnik JF, Brown DM, Chong V, Nguyen QD, et al. Intravitreal aflibercept injection for neovascular age-related macular degeneration: ninety-six-week results of the VIEW studies. *Ophthalmology.* 2014;121(1):193-201.

16.Brown DM, Heier JS, Ciulla T, Benz M, Abraham P, Yancopoulos G, et al. Primary endpoint results of a phase II study of vascular endothelial growth factor trap-eye in wet age-related macular degeneration. *Ophthalmology.* 2011;118(6):1089-97.

17.Heier JS, Boyer D, Nguyen QD, Marcus D, Roth DB, Yancopoulos G, et al. The 1-year results of CLEAR-IT 2, a phase 2 study of vascular endothelial growth factor trap-eye dosed as-needed after 12-week fixed dosing. *Ophthalmology.* 2011;118(6):1098-106.

18.Malik D, Tarek M, Caceres del Carpio J, Ramirez C, Boyer D, Kenney MC, et al. Safety profiles of anti-VEGF drugs: bevacizumab, ranibizumab, aflibercept and ziv-aflibercept on human retinal pigment epithelium cells in culture. *Br J Ophthalmol.* 2014;98 Suppl 1:i11-6.

19.Nguyen QD, Campochiaro PA, Shah SM, Browning DJ, Hudson HL, Sonkin PL, et al. Evaluation of very high- and very low-dose intravitreal aflibercept in patients with neovascular age-related macular degeneration. *Journal of ocular pharmacology and therapeutics* : the official journal of the Association for Ocular Pharmacology and Therapeutics. 2012;28(6):581-8.

20.Trichonas G, Kaiser PK. Aflibercept for the treatment of age-related macular degeneration. *Ophthalmology and therapy*. 2013;2(2):89-98.

21.Chhablani J. Intravitreal ziv-aflibercept for recurrent macular edema secondary to central retinal venous occlusion. *Indian journal of ophthalmology*. 2015;63(5):469-70.

22.Paulose R, Chhablani J, Dedhia CJ, Stewart MW, Mansour AM. Intravitreal ziv-aflibercept for macular edema following retinal vein occlusion. *Clin Ophthalmol.* 2016; 10: 1853-8.

23.Heier JS, Brown DM, Chong V, Korobelnik JF, Kaiser PK, Nguyen QD, et al. Intravitreal aflibercept (VEGF trap-eye) in wet age-related macular degeneration. *Ophthalmology.* 2012;119(12):2537-48.

24.Boyer D, Heier J, Brown DM, Clark WL, Vitti R, Berliner AJ, et al. Vascular endothelial growth factor Trap-Eye for macular edema secondary to central retinal vein occlusion: six-month results of the phase 3 COPERNICUS study. *Ophthalmology*. 2012;119(5):1024-32.
25.Brown DM, Campochiaro PA, Singh RP, Li Z, Gray S, Saroj N, et al. Ranibizumab for macular edema following central retinal vein occlusion: six-month primary end point results of a phase III study. *Ophthalmology*. 2010;117(6):1124-33.e1.
26.Chakravarthy U, Harding SP, Rogers CA, Downes SM, Lotery AJ, Wordsworth S, et al. Ranibizumab versus bevacizumab to treat neovascular age-related macular degeneration: one-year findings from the IVAN randomized trial. Ophthalmology. 2012;119(7):1399-411.
27.Martin DF, Maguire MG, Fine SL, Ying GS, Jaffe GJ, Grunwald JE, et al. Ranibizumab and bevacizumab for treatment of neovascular age-related macular degeneration: two-year results. *Ophthalmology*. 2012;119(7):1388-98.
28.Network TDRCR. Aflibercept, Bevacizumab, or Ranibizumab for Diabetic Macular Edema. *New England Journal of Medicine.* 2015;372(13):1193-203.
29.Wells JA, Glassman AR, Ayala AR, Jampol LM, Bressler NM, Bressler SB, et al. Aflibercept, Bevacizumab, or Ranibizumab for Diabetic Macular Edema: Two-Year Results from a Comparative Effectiveness Randomized Clinical Trial. *Ophthalmology*. 2016;123(6):1351-9.
30.Epstein DL, Algvere PV, von Wendt G, Seregard S, Kvanta A. Benefit from bevacizumab for macular edema in central retinal vein occlusion: twelve-month results of a prospective, randomized study. *Ophthalmology*. 2012;119(12):2587-91.
31.Holz FG, Roider J, Ogura Y, Korobelnik JF, Simader C, Groetzbach G, et al. VEGF Trap-Eye for macular oedema secondary to central retinal vein occlusion: 6-month results of the phase III GALILEO study. *Br J Ophthalmol*. 2013;97(3):278-84.
32.Rajagopal R, Shah GK, Blinder KJ, Altaweel M, Eliott D, Wee R, et al. Bevacizumab Versus Ranibizumab in the Treatment of Macular Edema Due to Retinal Vein Occlusion: 6-Month Results of the CRAVE Study. *Ophthalmic surgery, lasers & imaging retina.* 2015;46(8):844-50.
33.de Oliveira Dias JR, Xavier CO, Maia A, de Moraes NS, Meyer C, Farah ME, et al. Intravitreal injection of ziv-aflibercept in patient with refractory age-related macular degeneration. *Ophthalmic surgery, lasers & imaging retina.* 2015;46(1):91-4.
34.Chakravarthy U, Adamis AP, Cunningham ET, Jr., Goldbaum M, Guyer DR, Katz B, et al. Year 2 efficacy results of 2 randomized controlled clinical trials of pegaptanib for neovascular age-related macular degeneration. *Ophthalmology*. 2006;113(9):1508.e1-25.

35.Gagajewski A, Murakami MM, Kloss J, Edstrom M, Hillyer M, Peterson GF, et al. Measurement of chemical analytes in vitreous humor: stability and precision studies. *Journal of forensic sciences*. 2004;49(2):371-4.
36.Sturner WQ, Dowdey AB, Putnam RS, Dempsey JL. Osmolality and other chemical determinations in postmortem human vitreous humor. *Journal of forensic sciences*. 1972;17(3):387-93.
37.de Oliveira Dias JR, Badaro E, Novais EA, Colicchio D, Chiarantin GM, Matioli MM, et al. Preclinical investigations of intravitreal ziv-aflibercept. *Ophthalmic surgery, lasers & imaging retina*. 2014;45(6):577-84.
38.Kitchens JW, Do DV, Boyer DS, Thompson D, Gibson A, Saroj N, et al. Comprehensive Review of Ocular and Systemic Safety Events with Intravitreal Aflibercept Injection in Randomized Controlled Trials. *Ophthalmology*. 2016;123(7):1511-20.
39.Freund KB, Hoang QV, Saroj N, Thompson D. Intraocular Pressure in Patients with Neovascular Age-Related Macular Degeneration Receiving Intravitreal Aflibercept or Ranibizumab. *Ophthalmology*. 2015;122(9):1802-10.
40.Jabs DA, Nussenblatt RB, Rosenbaum JT. Standardization of uveitis nomenclature for reporting clinical data. Results of the First International Workshop. *Am J Ophthalmol.* 2005;140(3):509-16.

Appendix Ia- Case Definitions

A. Diabetic macular oedema (DME) is defined as macular oedema secondary to diabetic retinopathy. Patients with type 1 or 2 diabetes mellitus who present with foveal centre involving DME (defined as retinal thickening involving the 1 mm central sub- field thickness [CST]) on optical coherence tomography (OCT). Diabetes Mellitus diagnosis is based on: i. established history of diabetes mellitus that includes current anti-diabetic medication use(Insulin or oral hypoglycemic drugs). ii. Documented diabetes meeting any of following criteria,

a. Fasting plasma glucose level>126mg/dL
b. Non- fasting plasma glucose level >200mg/dl

B. **Retinal vein occlusion** (RVO) is defined as a blockage of the vessel which drains blood out of the retina, the light-sensitive tissue in the back of the eye. Macular oedema secondary to RVO must be foveal centre-involving. Criteria: Venous dilatation and tortuosity with scattered intra-retinal haemorrhages equal to or more than 1 disc area in one or more quadrant

and presence of retinal oedema on ophthalmoscopy for BRVO, or in all quadrants for CRVO, with or without exudates or cotton wool spots or optic disc oedema. The presence of vascular leakage and/or areas of capillary non-perfusion on fluorescein angiography is supportive evidence.

C. **Neovascular AMD** (nAMD) or choroidal neovascularisation (CNV) secondary to AMD is defined as neovascularisation arising from the inner choroid into the sub-RPE/subretinal space. Criteria for active nAMD include any of the following criteria:

i. Abnormal retinal thickness, particularly with evidence of intraretinal, subretinal, or sub pigment epithelial fluid accumulation, optimally confirmed by SD-OCT.
ii. Presence (or recurrence) of intraretinal or subretinal haemorrhage.
iii. New or persistent leakage shown on Fluorescein angiography.
iv. Choroidal neovascular membrane (CNV) enlargement on FA unless solely due to dry, fibrotic staining.
v. Visual acuity deterioration, considered likely to represent CNV activity.

APPENDIX I b- Statistical Plan

All statistical analyses for this study will be done using STATA 13 (Statacorp, Texas, USA). The frequencies of ocular and systemic adverse events and serious adverse events will be computed. Continuous variables will be presented as mean and standard deviation. Pre- and post-injection changes in BCVA, intra ocular pressure, and central subfield foveal thickness will be compared using paired t-test. ANOVA and MANOVA will be used to assess the repeated measures at times 0, 4, 8 and 12 weeks. A P value<0.05 will be considered statistically significant.

The unmasked statistician (EK) will analyse the data based on the following schedules:

1. After 50% of subjects have been recruited and followed for 1 month
2. After 100% of subjects have been recruited and followed up for 1 month.
3. After 100% of subjects have been recruited and followed up for 3 months

The interim analyses will include demographic and baseline characteristics by group, analysis of subjects screened, enrolled, active, completed and terminated, and primary outcome endpoints including the incidence of pain, blurred vision, raised intraocular pressure, cataract, intraocular inflammation, endophthalmitis, and systemic adverse events by group. The data will be presented by masked treatment groups to the data and safety monitoring board

(DSMB) and a decision on early stopping or continuation of the trial taken in the event of occurrence of large differences or serious adverse events observed in the interim analysis.

Appndix II- **INFORMED CONSENT FORM FOR PATIENTS WITH RETINAL VASCULAR DISEASES.**

Date:……….. Patient Code...........................**Age**........... **Gender**...M/F Study Site:……

STUDY TITLE: Safety of ziv-aflibercept in retinal diseases in a Ghanaian population

INSTITUTION	NAME OF PRINCIPAL INVESTIGATOR
Eye Centre, Korle-Bu Teaching Hospital	Dr. Imoro Zeba Braimah

1. **General Information**

   a. **The purpose of this research study:** We aim to compare two doses of ziv-aflibercept, 1.25mg and 2mg, to treat your eye condition to see which one is safe and works best. Ziv-aflibercept eye injection is an investigational product. An investigational product means the drug has not been approved yet by the Food and Drugs Authority of Ghana or any other drug regulating body as an eye injection.

   b. **The procedure of the study**: We will initially examine your eyes, take pictures of the back of your eye (Fundus photograph, optical coherence tomography and fluorescein angiography) with and or without the use of a dye which will be injected into a vein in your arm. You will be asked to undergo further test if necessary. You will be put into one of 2 groups (1.25mg dose versus 2mg dose) randomly. Random means by chance, like tossing a coin. You therefore have an equal chance of being in any of the 2 groups. To make sure the findings of this study are as accurate as possible, it is important that no one knows what dose of he/she is receiving until the end of the research. You will be given injection into your eye and we will see you 1 day and 7 days after the first injection to check your eye pressure and see if your eye is functioning well. You will be seen again at 4 weeks, 8 weeks and 12 weeks after the first injection. After the first injection, you will be given injection again at 4 weeks and 8 weeks. Your treatment is expected to go on for 3 months. The study will bear the full cost of your eye tests and injections**.**

   c. **The risks or dangers and discomforts from this study:** Injection into the eye is common for your eye condition in Korle- Bu Teaching Hospital (KBTH) and elsewhere. You are likely to have some discomfort after the

injection which will disappear after a few days. If you have pain that is persistent and associated will blurred vision please report immediately to this Eye Centre for examination because you may be developing infection in your eye. The chance of infection and other serious complication is less than 0.1%, which means less than one in one thousand people will get an infection. We will try to decrease the chances of infection and other dangers from happening by adhering to strict sterile rules during the injection. In the event of occurrence of eye pain, your eye will be examined to exclude raised eye pressure and swelling/infection in the eye, and the pain will be relieved with topical analgesic agents. In the presence of sterile swelling of your eye, you will be treated with corticosteroids eye drops and monitored daily for response to treatment. If an infection occurs we have the expertise to treat the infection and the treatment will be of no cost to you. About one-third of eyes who develop serious eye infection will have permanent loss of their vision despite treatment. All other complications that may occur will be treated at no cost to you.

d. **Expected Benefit of this study:** This injection has been used to treat patients with eye problems similar to yours in India, Lebanon, Egypt and Brazil. The available reports, although short term (3- 6 months) have shown that this injection is safe and resulted in improvement in vision. This injection has not been used in Ghanaians with similar eye condition. If the injection is found to be safe in this study, the information obtained will help improve the care given to Ghanaians with a similar eye condition. You have been insured against any serious side effects from the treatment. You will be given GHC5.00 for transportation per visit.
e. **Alternative treatments**: The use of eye injections is the present standard of care for your eye condition. There are two available eye injections- bevacizumab and ranibizumab. The safety and effectiveness of bevacizumab and ranibizumab are similar. Ranibizumab has been approved by the United States of America Food and Drugs Administration and European medicines agency for eye injections. Ranibizumab is highly expensive and less than 5% of our population use this drug. Bevacizumab is the most common eye injection for your condition because it is over 50 times cheaper than ranibizumab per injection (GHC400.00 versus GHC3000.00). The use of

bevacizumab injection is however, off-label, because it has not been approved by any regulatory authority for injection into the eye. Another treatment of your eye condition is with the use of laser. The use of laser for your eye condition is currently limited and usually does not result in improvement in vision.

2. **Confidentiality:** All information collected from this research will be kept confidential. By signing this written consent form, I grant permission to monitors, the ethics committee of KBTH, and the Food and Drugs authority (FDA) of Ghana for direct access to my medical records in order to verify the processes and/or data of this study without violating my confidentiality in accordance with the laws and regulations of Ghana. The findings of this study may be reported at meetings or in medical journals, but your name will not be used in the report. We will inform you of the outcome of this study on completion.
3. **Your Right to Refuse or Withdrawal of Participation:** Your participation in this research is voluntary. You have the right to withdraw at any time during this study without any penalty or impact on the treatment given you. You will not be refused treatment if you do not take part in this study or if you withdraw from this research at any Point.
4. **Regulation and Communication:**
    a. **Independent Contact person:** This proposal has been reviewed and approved by the KBTH Institutional Review Board (IRB). This is the committee whose job is to ensure that research participants are protected from harm. If you wish to ask someone independent about this research, please contact, IRB, Korle-Bu, Tel: 0302666766. This proposal has also been reviewed and approved by the FDA of Ghana.
    b. **Contact Information:** If you have any questions now or later, you may contact any of the following: Dr. IZ Braimah, Tel: 0206301363 and Dr. KN Amissah-Arthur, Tel:0276864343. If any new information regarding this drug that could affect your desire to continue participating in this study becomes available, we shall communicate to you immediately.

I(name) ……………………………………. of………………………have read the foregoing information, or it has been translated to me in a language I understand. I have had the opportunity to ask questions about it and answers given me are to my satisfaction. I grant permission to monitors, the Korle-Bu Teaching Hospital IRB, and the FDA of Ghana for direct access to my medical records in order to verify the processes and/or data of this study without violating my confidentiality in accordance with the laws and regulations of Ghana. I also grant permission to the principal investigator to use my data including the eye (retina) photographs and/ or images for publications without violating my confidentiality in accordance with the laws and regulations of Ghana. I consent voluntarily to participate as a subject in this study.

Signed..***Thumbprint (illiterate participant):***

Tel. No…………………….

In the presence of an independent literate/illiterate witness

signed……………………………… ***Thumbprint (illiterate witness):***

Date…………………………. Place/ Study Site
